# Inner Ear Drug Delivery for Sensorineural Hearing Loss: Current Challenges and Opportunities

**DOI:** 10.3389/fnins.2022.867453

**Published:** 2022-05-24

**Authors:** Sophie S. Liu, Rong Yang

**Affiliations:** ^1^Smith School of Chemical and Biomolecular Engineering, Cornell University, Ithaca, NY, United States; ^2^Meinig School of Biomedical Engineering, Cornell University, Ithaca, NY, United States

**Keywords:** drug delivery, inner ear, sensorineural hearing loss, small molecule, gene therapy, cell therapy

## Abstract

Most therapies for treating sensorineural hearing loss are challenged by the delivery across multiple tissue barriers to the hard-to-access anatomical location of the inner ear. In this review, we will provide a recent update on various pharmacotherapy, gene therapy, and cell therapy approaches used in clinical and preclinical studies for the treatment of sensorineural hearing loss and approaches taken to overcome the drug delivery barriers in the ear. Small-molecule drugs for pharmacotherapy can be delivered via systemic or local delivery, where the blood-labyrinth barrier hinders the former and tissue barriers including the tympanic membrane, the round window membrane, and/or the oval window hinder the latter. Meanwhile, gene and cell therapies often require targeted delivery to the cochlea, which is currently achieved via intra-cochlear or intra-labyrinthine injection. To improve the stability of the biomacromolecules during treatment, e.g., RNAs, DNAs, proteins, additional packing vehicles are often required. To address the diverse range of biological barriers involved in inner ear drug delivery, each class of therapy and the intended therapeutic cargoes will be discussed in this review, in the context of delivery routes commonly used, delivery vehicles if required (e.g., viral and non-viral nanocarriers), and other strategies to improve drug permeation and sustained release (e.g., hydrogel, nanocarriers, permeation enhancers, and microfluidic systems). Overall, this review aims to capture the important advancements and key steps in the development of inner ear therapies and delivery strategies over the past two decades for the treatment and prophylaxis of sensorineural hearing loss.

## Introduction

Hearing loss is the fourth leading cause of disability globally (Vos et al., 2017). It has been estimated that 466 million people, which represents over 5% of the global population, live with disabling hearing loss, defined as the inability to detect sound through vibrational mechanical energy or to convert it into electrochemical nerve signals. The latest estimate from 2021 by the World Health Organization puts the global economic burden of this disease at $980 billion (World Health Organization, 2021). Furthermore, hearing loss places immeasurable hindrances on a patient’s quality of life, as it has been shown to impede the development of speech, pose difficulties in social activities, or increase the risk of unemployment (Council, 2005; Cunningham et al., 2017).

**FIGURE 1:**
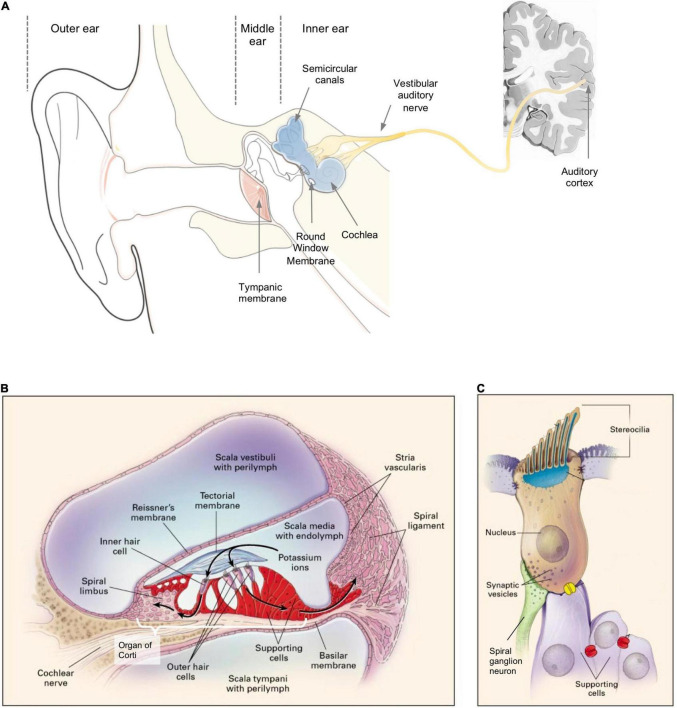
**(A)** A schema of the structure of an ear; sensorineural hearing loss (SNHL) is caused by lesions to the inner ear or neurons along the vestibular auditory nerve from the cochlea to the brain. **(B)** A cross-sectional schema of the cochlea showing the three scalae and associated anatomical structures. **(C)** A schema of a sensory hair cell. **(B,C)** Reprinted from Willems and Epstein (2000) with permission.

The anatomy of the ear can be divided into outer ear, middle ear, and inner ear (Figure 1A). The outer ear and the middle ear are separated by the tympanic membrane (TM). The middle ear contains three auditory ossicles which are responsible for sound transmission. The inner ear (also known as the labyrinth) houses the cochlea, the vestibule, and the semicircular canals. The cochlea is the auditory sensory organ responsible for hearing while the vestibule and the semicircular canals constitute the vestibular system which is responsible for balance and spatial orientation. Based on the ear anatomy, hearing loss can be classified into two types: (i) conductive hearing loss, which refers to hearing loss caused by lesions in the outer and middle ear, and (ii) sensorineural hearing loss (SNHL), which refers to hearing loss caused by lesions in the inner ear and the auditory nerve pathway (Figure 1A), hence requiring drug delivery to the inner ear (Cunningham et al., 2017). SNHL accounts for nearly 90% of all cases of hearing loss (Nyberg et al., 2019). It is also the most common sensory disease in developed countries (Smith et al., 2005). Here, we focus the discussion on SNHL and the inner ear drug delivery approaches that have been developed to address this disorder. We refer readers to existing reviews for detailed discussion on conductive hearing loss (Janssen et al., 2012; Dougherty and Kesser, 2015; Hill-Feltham et al., 2020).

Sensorineural hearing loss is characterized by the degeneration of two types of cells: cochlear hair cells, which are the primary mechanoreceptors for sound, and/or auditory nerve neurons, which transmit signals from the cochlea to the cochlear nucleus in the brainstem, the initial site of auditory processing (Møller, 2011; Cunningham et al., 2017). SNHL can have **non-genetic** and **genetic** etiology. Non-genetic factors include noise exposure (Daniel, 2007), viral infections (e.g., Zika, cytomegalovirus) (Cohen et al., 2014), chronic middle ear infection (English et al., 1973), ototoxic chemicals (e.g., chemotherapeutic drug cisplatin, aminoglycoside antibiotics) (Bisht and Bist, 2011), autoimmune disease (Mijovic et al., 2013), and aging (Gordon-Salant, 2005). There are also cases of idiopathic SNHL with no identifiable cause, termed sudden SNHL (SSNHL) and defined by the occurrence of a hearing loss of 30 dB or more within a window of 72 h (hr) (Kuhn et al., 2011). Around 70% of genetic SNHLs are **non-syndromic**, during which hearing loss occurs as the sole pathology. Non-syndromic hearing loss can be classified based on the gene loci into autosomal dominant (DFNA), autosomal recessive (DFNB), and X-linked recessive (DFN) which is less common than the other two (Willems and Epstein, 2000). Conversely, **syndromic** hearing loss occurs with a variety of additional clinical features. For example, Usher syndrome (USH1B/1F/1G/2A/3A) leads to symptoms of hearing loss and reduced capabilities of balance and eyesight.

Current treatment options approved by the United States Food and Drug Administration (U.S. FDA) for SNHL mainly comprise hearing aids and cochlear implants. Hearing aids are sound amplifiers worn around the outer ear and they are commonly prescribed to patients with mild to moderate hearing loss (Food and Drug Administration, 2018a). Cochlear implants are approved for use in patients with severe to profound hearing loss with surgical placement; they bypass the impaired ear structures and directly send sound-stimulated electric currents to the auditory nerve via the electrode placed in the cochlea (Food and Drug Administration, 2018b). Cochlear implants, when paired with intensive speech therapy, can help prelinguistically deaf children develop near-normal language skills (Clark, 2004). While high variabilities in patient outcomes have been reported for cochlear implants, these are likely due to insufficient simulation of natural hearing and the need for post-surgical cognitive rehabilitation (Zeng, 2016).

In clinical practice, corticosteroids, such as prednisone, prednisolone and dexamethasone, are recommended as first-line treatment by the American Academy of Otolaryngology to manage SSNHL (Chandrasekhar et al., 2019), although it has not yet been included in the FDA-approved indications. Oral corticosteroids are recommended within 2 weeks of onset of symptoms and intra-tympanic corticosteroids at 2–6 weeks if no recovery was observed. There exists considerable variability in the reported efficacy of corticosteroid therapy versus placebo. Furthermore, they are only effective within a short time window before permanent sensorineural damage sets in, and even when treated within this window, patients may not gain serviceable hearing from this therapy. These deficiencies have motivated exciting preclinical research that has focused on regenerative therapy to replenish hair cell and neuron population in the cochlea and restore their functions. For genetic SNHLs, which cannot be treated by traditional pharmacotherapy, gene therapies are being developed and tested in mouse models (Table 1); they aim to selectively replenish absent genes or correct defective genes to reinstate normal cochlear development and rescue hearing function.

**TABLE 1:** A list of genetic SNHL animal studies discussed in this review.

Gene	Locus/Syndrome	Mouse model	Gene therapy	Injection route, time at injection	Vector or non-viral carriers	Outcome	References
**Non-syndromic deafness**							
*Slc17a8*	DFNA25	Vglut3 knockout	Transgene delivery	Intra-RWM, P10–P12	AAV1 with CBA promoter	Transduction in 40% of IHCs; ABR threshold restored to near-normal level	Akil et al., 2012
*Otof*	DFNB9	Otoferlin knockout	Transgene delivery	Intra-RWM, P10, P19, P30	Dual AAV2 with CMV promoter	ABR thresholds (8–32 kHz) restored to near normal levels and maintained for about 30 weeks	Akil et al., 2019
*Tmc1*	DFNB7/11	Tmc1 knockout	Transgene delivery	Intra-RWM, P0–P2	AAV2/1 with CBA promoter	*Tmc1* mRNA expression in hair cells increased by 12-fold compared to unjected cochleae; slight ABR recovery at 8–16 kHz	Askew et al., 2015
	DFNB7/11	Baringo (*Tmc1 c.545A* > *G)*	CRISPR, cytosine base editor	Intracochlear, P1	AAV/Anc80L65 with Cbh promoter	∼30% reversion of mutant allele to wild-type *Tmc1* in sampled cochlear cells, ABR response at 5.6 kHz slightly recovered	Yeh et al., 2020
	DFNA36	Beethoven (*Tmc1 c.1235T* > *A*)	Transgene delivery	Intra-RWM, P0–P2	AAV2/1 with CBA promoter	Exogenous Tmc2 restored acoustic startle response threshold to 90 –100 dB, slight ABR recovery at 8–16 kHz	Askew et al., 2015
	DFNA36	Beethoven (*Tmc1 c.1235T* > *A*)	miRNA	Introcochlear, P0 - P2	AAV2/9 with CMV promoter	Expression of mutant allele suppressed by >88%; onset of hearing loss was delayed by ∼ 21 weeks	Shibata et al., 2020
	DFNA36	Beethoven (*Tmc1 c.1235T* > *A*)	CRISPR, NHEJ	Cochleostomy, P1	Lipofectamine 2000	IHC and OHC survival improved by ∼80% and ∼30%, respectively; slight decrease in ABR threshold compared to untreated mice	Gao et al., 2017
	DFNA36	Beethoven (*Tmc1 c.1235T* > *A*)	CRISPR, NHEJ	Intracochlear, P1	AAV/Anc80L65 with CMV promoter	Expression of mutant allele suppressed by 24% in cochlear cells; near normal ABR at 8 kHz but not higher frequencies	György et al., 2019b
*Gjb2*	DFNB1	Cx26 conditional knockout	Transgene delivery	Intra-RWM, P0	AAV1 with CMV promoter	*Gjb2* expression rescued in the supporting cells and partial restoration of ABR response	Iizuka et al., 2015
	DFNA3	*Gjb2 p.R75w transfection*	siRNA	Co-delivered onto a gel foam, placed outside the RWM, P42–P45	For transfection: CMV-driven mammalian expression vector complexed with GeneShuttle for siRNA: GeneShuttle cationic liposome	Mutant *Gjb2* allele expression suppressed by >70%, click ABR restored to similar level as healthy animal	Maeda et al., 2005
*Cdh23*	DFNB12, USH1D	*Cdh23 ahl (c.753G* > *A)*	CRISPR, HDR	Embryo microinjection	None	Restored normal OHC hair bundle count at the basal turn and reduced ABR threshold at 32 kHz by >25 dB	Mianné et al., 2016
**Syndromic deafness**							
*Clrn1*	USH3A	Clarin-1 conditional knockout	Transgene delivery	Intra-RWM, P2–P3	AAV2/8	Corrected stereocilia morphogenesis, partially preserved hearing sensitivity	Dulon et al., 2018
*Ush1c*	USH1C	*Ush1c (c.216G* > *A)*	Small antisense oligonucleotides	IP, P3–P5	None	Partially rectified the pre-mRNA splicing of *Ush1c*, restored harmonin production, lowered ABR threshold to near normal level at 8, 16 kHz	Lentz et al., 2013
	USH1C	*Ush1c (c.216G* > *A)*	Transgene delivery	Intra-RWM, P0 - P1	AAV2/Anc80L65 with CMV promoter	Recovery of mechanosensitivity in both OHCs and IHCs, ABR recovery to near normal levels at 5.6–16 kHz	Pan et al., 2017

In this review, we will discuss three classes of treatment options (divided based on the type of therapeutics being delivered): pharmacotherapy, gene therapy, and cell therapy (Table 2). Within each class, the discussion is further organized based on the delivery vehicles and delivery route used. To achieve efficacy, all three classes of treatment require overcoming one or more types of biological barriers in the ear. Below, we first review the structure of the inner ear in the context of SNHL, pointing out the target anatomic locations for drug delivery. Building from that, a brief overview of the potential barriers and common delivery approaches employed thus far to overcome these barriers will be presented at the end of this section.

**TABLE 2:** General pharmacotherapy, gene therapy, and cell therapy treatment strategies for SNHL.

Therapy	Types of SNHL targeted	Therapeutic cargoes	Common delivery vehicles	Common delivery routes
**Pharmacotherapy** administration of corticosteroids and otoprotectants	Non-genetic	Small molecule	Hydrogel nanocarriers adjuvants	Systemic, intra-tympanic, intracochlear, intralabyrinthine, cochlear implant, microfluidic device
**Gene therapy**				
**Transgene delivery** introduce foreign genetic material to replenish wild-type copies of the deafness-causing gene	Genetic	DNA plasmid	Viral vectors	Intracochlear, intralabyrinthine
**Gene silencing** suppress the expression of the mutant allele at transcriptional or translational level	Genetic	DNA plasmid siRNA, miRNA	Viral vectors non-viral nanocarriers	Intracochlear, intralabyrinthine
**Gene editing** permanently edit the deafness-causing gene in host DNA genome	Genetic	DNA plasmid nucleotides, protein	Viral vectors non-viral nanocarriers	Intracochlear, intralabyrinthine
**Gene delivery for cell regeneration** induce hair cell or SGN regeneration from endogenous tissue	Non-genetic	DNA plasmid nucleotides, protein	Viral vectors non-viral nanocarriers	Intracochlear, intralabyrinthine
**Cell therapy** transplantation of exogenous cells to restore auditory function or manage AIED	Non-genetic	Cell	n/a	Systemic, intracochlear, intralabyrinthine

### Structure of the Inner Ear and the Pathophysiology of Sensorineural Hearing Loss

The auditory sensory organ, cochlea, is spiral in shape and contains three chambers: the scala vestibuli (vestibular duct), the scala media (cochlear duct), and the scala tympani (tympanic duct) (Figures 1A,B; Raphael and Altschuler, 2003). The scala media is filled with endolymph fluid while the other two scalae are filled with perilymph fluid (Raphael and Altschuler, 2003). These fluids are maintained at specific ion compositions to facilitate the mechanoelectrical transduction of sound by the hair cells (Park, 2015).

The basilar membrane, which separates the scala media from the scala tympani, houses the organ of Corti (Figure 1B) – a sensory epithelium containing one row of inner hair cells (IHCs), three rows of outer hair cells (OHCs), and multiple rows of supporting cells. Each hair cell has a mechanosensing organelle called the stereocilia (Figure 1C) – made up by bundles of actin filaments – which respond to sound-induced shear in the endolymph fluid and stimulate depolarization of hair cells to release neurotransmitters to the spiral ganglion neurons (SGNs) (Petit et al., 2001). The tectorial membrane (Figure 1B) is a specialized extracellular matrix which is in direct contact with the OHCs at the apical surface and is believed to be involved in stereocilia deflection and calcium storage (Strimbu et al., 2019). The Reissner’s membrane separates the scala media and scala vestibuli (Figure 1B; Zou et al., 2016). The lateral wall of the scala media houses the stria vascularis and the spiral ligament which are both responsible for maintaining the resting potential and ion homeostasis of the endolymph. The symptoms observed in SNHL (e.g., shift in auditory threshold, absence of otoacoustic emission) could mask their heterogenous underlying etiology, which often varies case-by-case and spans multiple cellular and tissue structures in the inner ear.

Primary defects in non-genetic SNHL can involve degeneration of the hair cells and supporting cells in the organ of Corti, loss of the SGNs, and atrophy of the stria vascularis (Merchant et al., 2005; Kuhn et al., 2011). In some cases, occlusion of blood supply, rupture of cochlear membranes, or non-specific systemic inflammation can also be the cause of non-genetic SNHL (Le Prell et al., 2007; López-González et al., 2012). The targeted cell or tissue for steroid therapy, which is the most common treatment for SSNHL, still remains poorly understood albeit decades of clinical use (Trune and Canlon, 2012). Glucocorticoid receptors are present in most of the inner ear tissues (e.g., organ of Corti, stria vascularis, and spiral ligament), in circulating immune cells, in neurons of the central auditory nervous system, and in many other organs (Le Prell et al., 2007). As a result, the action site of glucocorticoid-induced gene transcription remains elusive in treating hearing disorders (Trune and Canlon, 2012).

**FIGURE 2:**
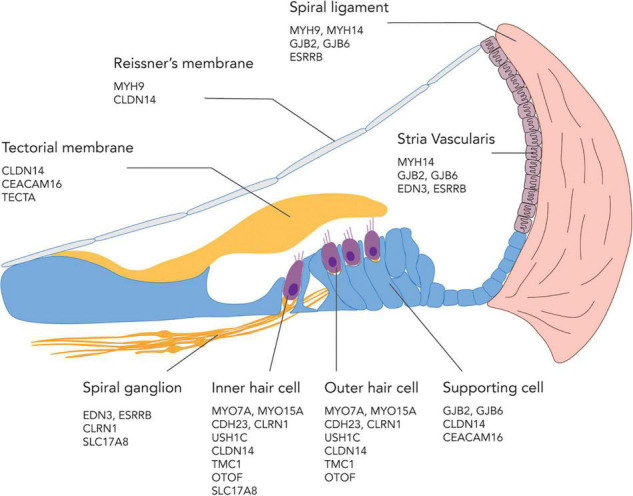
An illustration showing possible sites of genetic defects in the cochlea and a subset of genes involved in SNHL at each location.

The site of defects in genetic SNHL are also diverse, including but not limited to the IHCs, OHCs, and supporting cells in the organ of Corti, as well as the stria vascularis, spiral ligament, tectorial membrane, and the SGNs (Figure 2). For a comprehensive review on the gene ontology of hearing loss based on each cell type and location in the cochlea, see Nishio et al. (2015). The target of gene therapies is naturally the cell type that is affected by the mutation of interest, with IHCs, OHCs, supporting cells, and SGNs being the main focus in recent studies (Ahmed et al., 2017). In general, the cellular expression profile of causative genes in the inner ear has a low degree of overlapping. For example, *Cdh23*, which encodes an adhesion molecule cadherin, is only expressed in IHCs and OHCs but not in the supporting cells or SGNs. However, some genes, such as *Cldn14* which encodes a protein, claudin-14, that is required for tight junction, are expressed in almost every cells in the organ of Corti (Nishio et al., 2015). Gene therapies aiming to induce endogenous cell regeneration often target the supporting cells for transdifferentiation into hair cells or the SGNs for neuronal regeneration. Cell therapies usually rely on the transplanted cells to home to their native location in the cochlea. In the next section, different layers of tissue barriers to these sites of defect in the inner ear will be discussed.

### Biological Barriers in the Ear and Drug Delivery Routes

**FIGURE 3:**
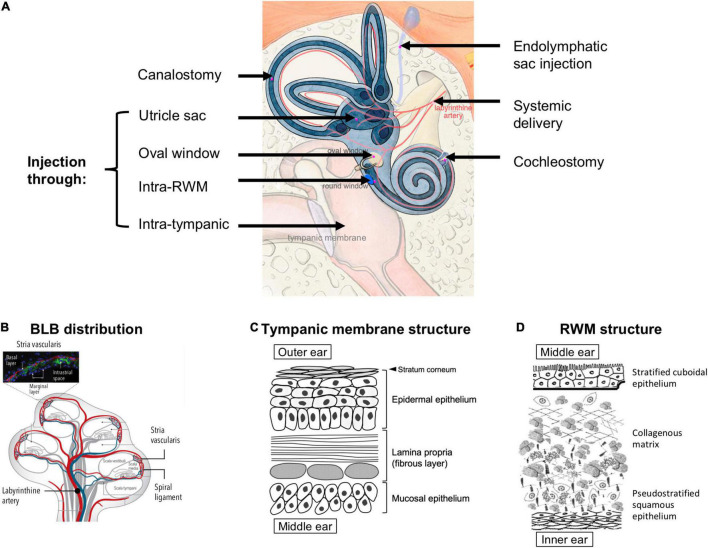
**(A)** Possible anatomical routes for therapeutic delivery into the inner ear; adapted from Delmaghani and El-Amraoui (2020), available under Creative Commons license (CC BY 4.0). **(B)** Distribution of the blood labyrinthine barrier (BLB), proposed in the literature based on existing experimental evidence; reprinted from Nyberg et al. (2019) with permission. **(C)** A schema of the structures and cell types in the tympanic membrane. **(D)** Structure of the round window membrane (RWM); adapted from Pyykkö et al. (2013), available under Creative Commons license (CC BY-NC 4.0).

There are several delivery approaches to overcome the anatomical barriers to the inner ear (Figure 3A). Systemic delivery has one of the easiest administration route but is hindered by the blood labyrinth barrier (BLB) which separates the inner ear fluids from blood circulation (Jahnke, 1980; Rybak et al., 2019). The exact anatomical location of BLB is poorly understood due to multiple possible origins of the perilymph and endolymph (Figure 3B; Bielefeld and Kobel, 2019; Nyberg et al., 2019). A well-characterized site of BLB is the stria vascularis (Figure 3B inset), which has a complex structure containing two epithelial layers separated by a narrow intrastrial space (Nin et al., 2008). Blood capillaries go through the intrastrial space, and these vessels are fenced off by endothelial cells. Tight junctions within the epithelial and endothelial linings provide layers of barrier between the blood, intrastrial space, and endolymph (Shi, 2016). In general, The BLB has several properties akin to those of the blood brain barrier (BBB) and similarly to the BBB, compounds which can permeate across the BLB are limited to small cationic molecules (Jahnke, 1980). Despite the similarity in physiological structure, uptake of several small molecules, e.g., salicylate, gentamicin, trimethylphenylammonium (TMPA), across the BBB is notably different from that across the BLB after systemic administration (Jastreboff et al., 1986; Inamura and Salt, 1992; Le and Blakley, 2017), prompting more studies to elucidate the difference in the transport mechanism across the two barriers (Nyberg et al., 2019).

To bypass the BLB, local delivery to the inner ear has been explored extensively. This approach often requires that the therapeutics first cross the barrier between the outer and middle ear, i.e., the TM, and then the barrier between the middle and inner ear, i.e., the round window membrane (RWM) or oval window (Figure 3A). The TM is a three-layered membrane containing the stratum corneum that is non-penetrable by most drugs (Figure 3C). Recent research has demonstrated the possibility of delivering therapeutics across intact TM using trans-tympanic platforms that temporarily enhanced the permeation of therapeutics without the need for piercing the TM (Yang et al., 2016, 2019). However, considerable future research will be required to render this non-invasive approach appropriate for treating inner ear diseases, because the concentration of therapeutics that permeate across intact TM can decrease by up to 100-fold (Yang et al., 2018). To achieve greater drug concentrations in the middle ear, intratympanic injection has been widely used in outpatient clinics. This procedure is regarded as safe and easy to perform despite a small risk of permanent TM perforation. From thereon, the drug needs to traverse across the RWM or the oval window to access the inner ear (Zhang et al., 2020).

The RWM is made up by three layers: an epithelial layer facing the middle ear, a connective tissue layer in between, and an epithelial layer facing the scala tympani of the cochlea (Goycoolea and Lundman, 1997; Figure 3D). The RWM is semi-permeable and drug diffusion is usually in favor of those with low molecular weight, high lipid solubility, and positive charge (Swan et al., 2008). Cationic molecules can more easily pass through the RWM because cell surfaces display many anionic proteins (Ikeda and Morizono, 2016). It has been hypothesized that small substances (<1000 g/mol) enter the RWM through passive diffusion while larger substances (>10,000 g/mol), such as albumin and horseradish peroxidase, rely on pinocytosis for active transport across the RWM (Goycoolea et al., 1987, 1988; Juhn et al., 1988). The RWM structure can be temporarily altered due to inflammatory events, e.g., otitis media has been shown to increase human RWM thickness from 70 μm to 89–114 μm and induce higher vascular permeability (Sahni et al., 1987). Several adjuvant agents, such as histamine, hyaluronic acid, pontocaine, or endotoxin, have been used to temporarily increase RWM permeability without causing long-term damage (Saber, 2010; Creber et al., 2019). For drugs that have crossed the RWM, there may exist a gradient of concentration in the perilymph, with the highest concentration at the basal end of the cochlea and the lowest at the apex (Creber et al., 2019). Since the longitudinal flow rate of the perilymph is extremely low (1.6 nL/min in the scala tympani) (Ohyama et al., 1988), drug transport is solely reliant on simple diffusion.

The oval window is closed by the stapes footplate and it separates the scala vestibuli from the middle ear (Figure 3A). Drug delivery through the oval window is less well studied than that through the RWM. Nevertheless, a few substances, such as the gadolinium, TMPA, gentamicin, chitosan nanoparticles, have been shown to use this route to enter the perilymph through the stapes (King et al., 2011, 2013; Salt et al., 2012; Ding et al., 2019).

Compared to the aforementioned permeation-based trans/intra-tympanic delivery approaches, intra-cochlear or intra-labyrinthine delivery approaches (Figure 3A) target the inner ear more directly with a precisely controlled amount of therapeutics. However, they require more invasive surgical procedures and carry the risk of disrupting the separations between the endolymph (∼150 mM potassium and ∼5 mM sodium) and perilymph (∼10 mM potassium and ∼140 mM sodium), which may lead to temporary dysfunction of the ion composition-dependent mechanotranduction (Park, 2015). Moreover, since the perilymph drains into the cerebrospinal fluid through a small canal termed the cochlear aqueduct, delivering therapeutics directly to the inner ear carries an additional risk of introducing these therapeutics, which may be in the form of small molecule drugs, proteins, or viral vectors, to the central nervous system (Salt et al., 2016; Salt and Hirose, 2018). Another potential route for intra-tympanic substances to reach the brain is through the vestibular auditory nerve (Zhang et al., 2012). Nonetheless, direct access into the cochlea may be preferred for the delivery of gene therapy and cell therapy, for which the barriers of TM, RWM, oval window, bony otic capsule, and the BLB may prove challenging to overcome (Plontke et al., 2016).

Intra-RWM injection is a type of intra-cochlear delivery, which pierces through the RWM to deliver the therapeutics into the scala tympani. It is associated with less surgical complications compared to other strategies (e.g., drilling through the bony otic capsule). The therapeutics delivered via intra-RWM need to cross the basilar membrane (Figure 1B), which separates the scala tympani and the scala media, to access the organ of Corti and achieve efficacy. Since the basilar membrane is an endothelial lining filled with tight junctions, it represents a permeation barrier to most drugs.

To gain direct access to the organ of Corti, cochleostomy can be used. The surgical procedure creates a separate opening to the lateral wall of the cochlea (Figure 3A), thus enabling direct access to the scala media (e.g., for delivering viral vectors to sensory cells through the apical side in treating genetic SNHL) (Stöver et al., 1999; Adunka et al., 2007). Cochleostomy can also be used to access the scala tympani for drug delivery. In addition, a number of drug-impregnated cochlear implants (Wilk et al., 2016) and pump systems with continuous or reciprocating infusion (Tandon et al., 2016) have been developed to enable long-term infusion of small molecules and proteins into the scala tympani.

Intra-labyrinthine delivery approaches can also provide direct access to the endolymph. They include canalostomy, which involves opening of the posterior semicircular canal, utricle injection and endolymphatic sac injection (Delmaghani and El-Amraoui, 2020; Figure 3A). These methods may be used to deliver therapies targeting vestibular hair cells or for therapies targeting cochlear cells while avoiding potential surgery-related hearing loss from cochleostomy (Guo et al., 2018a; Lee et al., 2020).

In the upcoming sections of this review, the discussion will be organized into three topics based on the nature of the therapeutic cargo. The first topic will be dedicated to the delivery of small-molecule steroids or otoprotective agents, which can be administered through systemic, minimally invasive, or direct routes; the second topic will focus on the delivery of large biomacromolecules for gene therapies and the third topic on the transplantation of stem cells and stem cell-derived progenitor cells. Biomacromolecules and cells are largely restricted to intra-cochlear and intra-labyrinthine delivery due to their large size and susceptibility to degradation.

## Pharmacotherapy and Delivery Methods

### Systemic Delivery

Systemic infusion of small-molecule drugs is mostly impeded by the BLB to reach the inner ear. Nevertheless, a selection of small molecules has been identified, which can cross the BLB effectively. In one example, a tracer (TMPA, 136 g/mol, logP = 1.8) that was intravenously (i.v.) injected into guinea pig was detected in the perilymph of the scala tympani and scala vestibuli after 90 min at 6.3 and 3.7% of the plasma concentration, respectively (Inamura and Salt, 1992). A number of ototoxins have also been discovered to cross the BLB. Aminoglycoside antibiotics, e.g., gentamicin (477 g/mol), kanamycin (484 g/mol), could cause cochleo- and vestibulo-toxicity when taken orally (Forge and Schacht, 2000). Cisplatin (300 g/mol), an antitumor drug, also has high cochleo-toxicity when administered systemically, causing irreversible hearing loss in 60% of treated patients (Karasawa and Steyger, 2015). Both aminoglycoside and cisplatin are able to enter the endolymph through the stria vascularis following systemic administration (Bunting et al., 2004; Li and Steyger, 2011), implying that these hydrophobic and cationic molecules with molecular weight ranging from 300 to 600 g/mol could penetrate the BLB. The exact mechanisms are not known but studies have suggested that the ion channels and cell transporters of the marginal cells in the stria vascularis aid the transportation of these drugs (Kros and Steyger, 2019). During an infection, drug penetration across the BLB tend to increase due to vasodilation that increases the permeability of capillaries and infiltration of inflammatory cells and factors into BLB (Sun and Wang, 2015).

In some cases, otoprotective agents are administered systemically to counteract the ototoxins. For example, sodium thiosulfate (STS, 158.11 g/mol, logP = –4.35), when administered i.v. a few hours after intracerebral infusion of cisplatin, delayed the onset of ototoxicity (Doolittle et al., 2001). In a phase III trial, concurrent intra-arterial injection of STS with cisplatin reduced the ototoxicity of cisplatin, as manifested by a reduction of the fraction of patients needing hearing aids from 49 to 36%. The concurrent injection did not negatively impact the locoregional tumor control rate or the overall patient survival (Zuur et al., 2007). This chemoprotectant exerted its effect without necessarily crossing the BLB, but by scavenging cisplatin in systemic circulation. Recently, a different class of otoprotectant, Dabrafenib (520 g/mol, LogP = 2.9) – a BRAF kinase inhibitor, was shown to cross the BLB after oral gavage in adult mice and protected the post-mitotic cochlea from cisplatin- and noise-induced hair cell death (Ingersoll et al., 2020). The exact mechanism for Dabrafenib’s penetration across the BLB has not been revealed, but its unique otoprotective property is conditional on its translocation into the cochlea to counteract cisplatin-induced BRAF signaling cascade and hair cell death (Ingersoll et al., 2020).

Systemic administration of corticosteroids have been prescribed in the clinic for otologic management of a variety of non-genetic SNHLs, including those induced by acoustic trauma (Chang et al., 2017) and ototoxin (Marshak et al., 2014), SSNHL (Schreiber et al., 2010), and autoimmune inner ear disease (AIED) (Buniel et al., 2009; Schreiber et al., 2010). The exact mechanism behind corticosteroids’ otoprotective actions is not known but suspected to be related to their well-known anti-inflammatory and immunosuppressive effects, or the promotion of blood supply to the inner ear (Chen et al., 2003; Trune and Canlon, 2012). Current standard of treatment for SSNHL recommends a short course (∼10 days) of oral corticosteroids (e.g., dexamethasone, prednisone, and prednisolone) with taper (Schreiber et al., 2010). In a 10 years retrospective study in the U.S., significant improvement in hearing (*p* < 0.01) was noted for patients with severe SSNHL treated with oral corticosteroid (prednisone, 60 mg/day) compared to placebo; however, no significant improvement was seen for patients with mild-to-moderate hearing loss (Chen et al., 2003). A systemic review of prospective, randomized trials concluded that the value of oral steroid treatment for SSNHL remains elusive due to conflicting results and the small number of clinical cases (Wei et al., 2013).

A downside of systemic delivery is the potential side effects or dose-limiting toxicity. Clinically, systemic use of high-dose corticosteroids has been associated with adrenal suppression, osteoporosis, hyperglycemia, weight gain, and gastritis (Stachler et al., 2012; Liu et al., 2013). A prospective study with 116 AIED patients treated with prednisone (60 mg/day, 1 month with taper) noted adverse events in 16 patients (14%), with the most common condition being hyperglycemia (discovered in nine patients, 7.8%); seven patients had to discontinue the corticosteroid regimen due to adverse events (Alexander et al., 2009). In an acoustic trauma mouse model, the systemic administration of a small-molecule γ-secretase inhibitor was unsuccessful in promoting hearing recovery because the high dose required to achieve therapeutic effect (50 mg/kg) reportedly led to significant side effects (not specified in the report) (Mizutari et al., 2013). Local injection of this drug through the RWM overcame the systemic side effects, inhibited Notch signaling in the organ of Corti, and induced hair cell regeneration via transdifferentiation of supporting cells (Mizutari et al., 2013).

### Intra-Tympanic Delivery

Intra-tympanic injection is a local, minimally invasive route which delivers the therapeutics directly into the middle ear for subsequent diffusion into the inner ear. Compared to systemic infusion, steroids injected via this route have been proposed to be more effective in treating noise- or ototoxin-induced hearing loss and SSNHL while reducing the systemic side-effects (Xenellis et al., 2016; Rybak et al., 2019). Drugs can be prepared as simple solutions, or formulated with adjuvants to increase drug permeation across RWM (Saber, 2010; Creber et al., 2019; Zhang et al., 2020), or encapsulated in nanocarriers or polymer matrices to extend drug release.

#### Simple Solutions

In a small clinical trial, methylprednisolone administered through intra-tympanic injection improved hearing sensitivity and speech discrimination for a subset of SSNHL patients (4/20) who failed to respond to oral steroids (Slattery et al., 2016). In another study, concentration of methylprednisolone in the perilymph was shown to be 126-fold higher following an intra-tympanic injection (40 mg) compared to that after i.v. injection (1 mg/kg) in human patients (Bird et al., 2007). Intra-tympanic dexamethasone also resulted in significant improvement in hearing sensitivity in a retrospective clinical study of 10 patients (Chandrasekhar, 2001). However, in a multi-center clinical trial with 250 unilateral SSNHL patients, intra-tympanic delivery of methylprednisolone (10 mg/day × 4 dose over 14 days) yielded similar hearing recovery as the orally administered prednisone (60 mg/day × 14 days, 5 days taper) (Rauch, 2011). Prednisone is only available in oral formulations and thus a similar corticosteroid, methylprednisolone, which is available in injectable form and slightly more potent, was used for intra-tympanic delivery, which could have contributed to the observed results. As such, the superiority of intra-tympanic steroid over oral steroid remains inconclusive (Stachler et al., 2012).

In a preclinical study, intra-tympanic administration of dexamethasone (0.12–0.168 mg/ear) has been shown to reduce cisplatin-induced ototoxicity (14 mg/kg intraperitoneal cisplatin) in 1–2 months old mice (Hill et al., 2008). The administration preserved auditory brainstem response (ABR) threshold to near-normal levels at lower frequencies (8 and 16 kHz) although the ABR threshold only increased by 20 dB at 32 kHz. In a cisplatin-treated (12 mg/kg intraperitoneal cisplatin) guinea pig model, similar otoprotective effect has been shown for intra-tympanic dexamethasone (0.4–1.2 mg/ear), the administration of which preserved the distortion product otoacoustic emissions (DPOAEs) amplitudes at 1–6 kHz to near-normal levels prior to cisplatin injection (Daldal et al., 2007).

Physical devices have also been designed to set up a conduit from external ear to the cochlea for drug infusion. The Silverstein MicroWick™ is a small catheter made by polyvinyl acetate, surgically inserted through an opening made on the TM to be placed just outside the RWM. One end of the catheter is accessible from the external ear canal to enable self-administered gentamicin for vertigo alleviation (Hill et al., 2006) or methylprednisolone for treatment of SSNHL (van Wijck et al., 2009). Drug application through MicroWick™ showed improvements in clinical outcome among 53 out of 69 MD patients for managing vertigo symptoms (Hill et al., 2006) and among 8 out of 12 SSNHL patients for improving the pure tone average response (van Wijck et al., 2009).

Refractory Ménière’s disease (MD) has also been managed clinically via intra-tympanic injection of corticosteroids, e.g., dexamethasone and methylprednisolone, based on their anti-inflammatory effects (Patel et al., 2016; Nevoux et al., 2018). Treatment of MD with severe vertigo sometimes resorts to intra-tympanic injection of gentamicin, termed transtympanic gentamicin (TTG) in clinical settings, despite the drug’s potential ototoxicity (Harner et al., 2001; Suryanarayanan et al., 2008).

#### Therapies With Enhanced Permeation

Based on the hypothesis that diffusion across the RWM or oval window is the rate-limiting step in intra-tympanic-injection, recent research efforts have focused on delivery modalities designed to increase the membrane permeability.

Several adjuvant agents are known to temporarily upregulate RWM permeability. For example, dexamethasone co-administered with histamine onto a gelatinous hyaluronic acid disk placed outside the RWM showed enhanced drug penetration compared to dexamethasone-alone in guinea pigs (Creber et al., 2019). A few known chemical permeation enhancers, including benzyl alcohol, saponin, and *N*-methyl-2-pyrrolidone (NMP), have also been shown to increase the permeation of fluorescent dexamethasone into the perilymph in guinea pigs, likely through disrupting the lipid bilayers of the RWM (Li et al., 2018).

Nanocarriers, including liposome, micelles, polymeric nanoparticle, and dendrimers which are less than 1 μm in size, have emerged recently as promising delivery vehicles to permeate the RWM. These nanocarriers can mask the physical characteristics of their payloads and thus increase drug stability and permeability. Some nanocarriers can translocate across the RWM through active transport. For example, PLGA nanoparticles (NP) were reported to have permeated the RWM via transcellular pathways instead of paracellular pathways; they entered the epithelial cells predominantly through macropinocytosis and caveolin-mediated endocytosis, and were degraded by the digestive endolysosomal pathway and/or secreted via exocytosis (Zhang et al., 2018). In a guinea pig model, PLGA NPs with the diameter of 140–180 nm were placed outside the RWM and shown to enter the perilymph more effectively than systemically delivered PLGA NPs (Tamura et al., 2005). Liposomes (diameter = 85 nm) and polymersome (diameter = 90 nm) NPs have also been reported to successfully carry a neurotoxic agent, disulfiram, across intact RWM in mice (Buckiová et al., 2012).

Superparamagnetic iron oxide NPs (SPION) were placed onto RWM and magnetized by an external magnetic field for directed entry into the perilymph (Ge et al., 2016). Although SPION itself cannot carry a payload, it has been encapsulated by drug-loaded PLGA to form a composite NP for targeted delivery. Interestingly, transmission electron microscopy (TEM) results suggested that SPION-PLGA composites (diameter 160–280 nm) were distributed throughout the inner ear with and without magnetic intervention. These NPs likely crossed the RWM via simple diffusion along the concentration gradient. Another magnetic NP was developed by Otomagnetics, Inc. (Ramaswamy et al., 2017). Methylprednisolone loaded into this magnetic NP was placed intra-tympanically into the middle ear of mice treated with cisplatin (4 mg/kg daily for 10 days in total). The drug-loaded magnetic NPs were directed through the RWM by a 0.5-Tesla external magnetic field. Magnetic delivery significantly reduced the incidence of hearing loss (53% at 32 kHz) compared to intra-tympanic injection only (97% at 32 kHz) or saline control (93%), and reduced cisplatin-induced cytotoxicity in the OHCs by 3.6-fold compared to intra-tympanic injection and by 7.2-fold compared to saline. This magnetic treatment caused reversible localized inflammation in the middle ear and no adverse safety issues (Shimoji et al., 2019).

#### Formulations With Prolonged Drug Release

The total amount of therapeutics in the inner ear can also be increased by prolonging the period over which active drug permeation across the RWM/oval window takes place, e.g., by increasing the residence time of the formulation in contact with the RWM (El Kechai et al., 2016).

Hydrogels are frequently used to prolong the release of small molecules and macromolecules. Criteria for a suitable hydrogel system for intra-tympanic delivery include low tissue toxicity or immunogenicity, biodegradability, mechanical tunability and sustained drug release profile. Fibrin, a hydrogel naturally derived from fibrinogen and thrombin, was used as a sustained-release vehicle for intra-tympanic gentamicin in chinchilla models (Balough et al., 2016). After a single dose injection of 2.5 mg gentamicin into the middle ear, gentamicin was detected in the perilymph for 72 hr at concentration above 50 μg/ml and not detected at all in the blood. The fibrin glue was also used in a prospective clinical study to treat MD patients (Casani et al., 2016). A single injection of 6 mg gentamicin into the middle ear was able to reduce the clinical signs of MD in 22/26 patients.

Dexamethasone loaded in silk fibroin-polyethylene glycol (PEG) hydrogel, applied onto the RWM of guinea pigs, helped maintain measurable drug concentration in the perilymph (100 ng/ml) for over 10 days and showed complete degradation of the hydrogel in 21 days (Yu et al., 2016). Likewise, dexamethasone impregnated in a poloxamer formulation, OTO-104, showed prolonged release for over 3 months in guinea pigs after intra-tympanic injection (Piu et al., 2011). This formulation was later marketed as Otividex by Otonomy Inc. Results from a phase III trial with 148 MD patients showed that a single intra-tympanic injection of Otividex did not effectively reduce the number of days of vertigo measured at 3 months compared to placebo (*p* = 0.312) (Taylor, 2021).

Hydrogels have been combined with NPs to endow sustained release kinetics to the nanocarriers. For example, interferon (IFN)-loaded PLGA NPs (diameter = 290 nm) were incorporated into a thermosensitive hydrogel composed of chitosan and glycerophosphate (Dai et al., 2017). The IFN-NP-hydrogel composite underwent sol-gel transition after intra-tympanic injection in guinea pigs and increased the IFN mean residence time in the perilymph by 3.2-fold compared to IFN solution, by 1.3-fold compared to IFN-PLGA, and by 1.6-fold compared to IFN-hydrogel. In another study, fluorescently labeled liposomes (diameter = 160 nm) were loaded into a chitosan hydrogel to increase the contact time with mice RWM, which led to the presence of liposomes in the perilymph and the cellular structures in the scala media 24 h after the injection (Lajud et al., 2015). Likewise, liposomes (diameter = 145 nm) with a dexamethasone prodrug were incorporated into a hyaluronic acid hydrogel for a single intra-tympanic injection in guinea pigs (El Kechai et al., 2016). The formulation enabled sustained release of dexamethasone in the perilymph of treated animals for 30 days after the injection (>25 ng/mL). Compared to prodrug loaded directly in the hydrogel, which achieved a maximum concentration of 39 ng/mL in the perilymph on 2-day post-injection, the liposomal hydrogel formulation increased the maximum concentration of dexamethasone to 833 ng/mL on 15-day post-injection. Confocal imaging revealed that a large proportion of these liposomes were trapped within the RWM – which the authors hypothesized to have acted as drug releasing reservoirs, and a small proportion of liposomes crossed the RWM intact (El Kechai et al., 2016).

### Intra-Cochlear and Intra-Labyrinthine Delivery

Injection through the RWM is a common delivery route adopted in the clinic as it is compatible with many drug formulations. Compared to systemic administration, this approach offers more effective targeting to the inner ear organ with lower dosage requirement. For example, intra-RWM injection of a small molecule-based γ-secretase inhibitor dissolved in PEG400 in neonatal mice lowered the required dose for significant induction of hair cell regeneration (*p* < 0.05) to 0.192 mg/ear from the 50 mg/kg used in systemic administration (Mizutari et al., 2013).

#### Intra-Cochlear and Intra-Labyrinthine Injection

Clinically, intra-cochlear injections are mostly used for preoperative or perioperative steroid regimens accompanying the surgical insertion of cochlear implant electrodes. A cochlear implant electrode is a neuroprosthesis used in patients who are severely hard-of-hearing to simulate hearing through stimulation of the auditory nerve. This device can be inserted through the RWM, or via a cochleostomy near the RWM (Richard et al., 2012). The immediate lesion and secondary lesion caused by the electrode insertion could be alleviated by steroid injections, which also lowers the inflammatory and fibrotic response due to surgical trauma, thus improving electrode-nerve interaction and protecting residual hearing (Paasche et al., 2006a).

In a prospective study conducted in 26 patients, injection of triamcinolone crystal (40 mg/ml) into the scala tympani before the electrode placement significantly lowered the intracochlear impedance over the first month (*p* < 0.05) compared to patients who did not receive the injection, suggesting better signal transmission between the electrode and inner ear tissues as a result of the steroid injection (Paasche et al., 2006a). In another study, however, triamcinolone delivered into 5 patients via a cochlear catheter prior to cochlear implant placement showed no significant difference in impedance (Prenzler et al., 2018). Due to limited numbers of clinical study available and large variations in study design and outcome measurement, currently, there is no clear consensus on the effectiveness of using preoperative and perioperative steroids in human for hearing preservation after cochlear implant (Santa Maria et al., 2014; Kuthubutheen et al., 2016; Snels et al., 2019).

#### Intra-Cochlear and Intra-Labyrinthine Delivery With Mechanical Devices

Research in the pre-clinical stage has sought to leverage the cochlear implant electrode itself as a drug-eluting reservoir to alleviate the complications of cochlear placement, including deterioration in residual hearing, inflammation, and fibrosis. Many groups have coated the electrodes with dexamethasone, released in a sustained fashion, to replace the existing perioperative corticosteroid injections. To list a few examples, silicone electrodes coated with 1 or 2% dexamethasone showed statistically significant improvements in residual hearing compared to an uncoated electrode in gerbils (*p* < 0.05 at frequencies 0.5–16 kHz after 4–6 weeks post-implantation) (Douchement et al., 2014) and guinea pigs (*p* < 0.05 at frequencies 8–24 kHz after 12–24 weeks post-implantation) (Liu et al., 2015a). A follow-up study showed that a 10% dexamethasone-coated electrode had a burst release profile of dexamethasone *in vivo* in the perilymph of guinea pigs (>1000 ng/ml in the first 1.5 h), and the release was sustained (>100 ng/mL) for 1 week (Liu et al., 2015b).

Promising results from many small animal studies show dexamethasone-coated electrode’s protective effect on residual hearing and anti-fibrotic property (Wilk et al., 2016), despite some controversy (Stathopoulos et al., 2014). Recently, a dexamethasone-eluting electrode was tested in ten non-human primates (macaques) with normal hearing (Manrique-Huarte et al., 2020). All animals experienced ABR thresholds shift after implantation, indicative of auditory damage, and at 6 months, the dexamethasone-eluting electrode group showed significantly decreased level of electrode impedance (*p* = 0.005), although the ABR threshold difference was not statistically significant (*p* = 0.37), between drug-eluting and non-eluting electrodes.

In addition to cochlear implant coating which permits passive drug elution, many pumping systems have been developed, alone or hybridized with cochlear implants, to enable a more active and controlled drug delivery option for the inner ear. An external pump connected to a microcatheter was tested in 23 patients who suffered from acute SNHL and failed systemic corticosteroid therapy to receive a local delivery of steroids for 4 weeks (Plontke et al., 2005). The microcatheter was surgically implanted into the posterior bony canal. Patients received methylprednisolone (40 mg/ml, 10 μl/h, *n* = 6) or dexamethasone (4 mg/ml, 5 μl/h, *n* = 17). At 3 weeks after the start of therapy, mean ABR threshold improved by 15 dB from 103 dB to 87 dB, showing a significant improvement compared to a historical control group (*p* < 0.001) who failed systemic corticoid and did not receive salvage treatment. This improvement was maintained at 1-year follow-up (Plontke et al., 2005).

Osmotic pumps have also been tested in animal models for continuous drug infusion into the inner ear but has not been applied to clinical study yet. For example, dexamethasone (100 ng/ml) delivered through a mini-osmotic pump connected to a cannula that was surgically implanted into the perilymph in noise-deafened guinea pigs attenuated ABR threshold shift by 10–20 dB (Takemura et al., 2004). In another study, guinea pigs with kanamycin-induced deafness were treated with dexamethasone infusion (1 ng/ml) through a microcannulation osmotic pump system (Himeno et al., 2002). The treated animals demonstrated a ∼ 20 dB shift in their ABR threshold and significantly higher OHC survival compared to untreated animals.

Implantable peristaltic pump has also been studied *in vitro* for the drug release and pharmacokinetic profile. Such pump system was first tested in guinea pigs for delivering a model drug, FITC-Dextran at concentrations of 40 mg/ml with 0.6 μl/h flow rate or at 4 mg/ml with 6 μl/h. The implantable peristaltic pump (iPRECIO) was connected to a cochlear implant electrode with an inbuilt cannula and surgically placed in the subcutaneous space of the animal (Sokolowski et al., 2017). Perilymph sample retrieved at 2, 24 h, and 7 days revealed a longitudinal gradient with higher drug concentration measured at the base compared to the apex, which persisted throughout the study period. This electrode-pump system was later studied in macaques with similar settings to measure the pharmacokinetic profile of FITC-Dextran (40 mg/ml, 2 μl/h) (Manrique-Huarte et al., 2021). Results from this study revealed that similar drug concentrations from the base to apex in the perilymph was achieved within 2–24 h after infusion, and the uniform drug distribution was maintained for 7 days. Discrepancies between these two studies provide a good example of how the anatomical and physiological differences between species, and likely between individuals too, could lead to variable pharmacokinetics in the cochlea.

The Contour cochlear implant electrode has also been modified to incorporate a drug delivery channel connected to a mechanical pump (Paasche et al., 2006b). Testing in a cochlear-shaped plastic model showed that drug distribution was diffusion-driven at low drug flow rate (1 μl/h) and flow-driven at high flow rate (100 μl/h). Another commercial cochlear implant (MED-EL PULSAR) has been modified to include a drug eluting channel connected to an external infusion pump (Hochmair et al., 2016). *In vitro* testing showed no septum leakage and fluid flow was maintained at 5 μL/h. Furthermore, a multichannel electrode array with polyimide tubing has been designed for connecting an external osmotic pump to the implant. This pump was tested in normal-hearing guinea pigs (Shepherd and Xu, 2002). Neomycin was perfused into the electrode with a flow rate of 0.25 μl/h for over 28 days. The electrode assembly remained patent with no breakage at the end of 28 days.

Compared to the aforementioned continuous perfusion systems which allow one-way flow, reciprocating perfusion systems can recirculate inner ear fluids through a valved drug reservoir to enable zero-net flow in the cochlea, thus achieving higher drug delivery rate and potentially lower frequency of drug refills (Sewell et al., 2009). The perilymph volume in humans is estimated to be about 160 μl, making it challenging to accommodate additional fluid in the cochlea in large volumes (Buckingham and Valvassori, 2016). Instead of introducing additional fluid into the cochlea, a reciprocating microfluidic device recycles the cochlear fluid to achieve continuous delivery. At the tested infusion rates (8.6–21 μL/min), drug distribution kinetics of this reciprocating perfusion system was also reliant on diffusion, based on evidence from computational modeling in guinea pig cochlea using a small molecule hair cell neurotransmitter blocker (6,7-dinitroquinoxaline-2,3-dione) (Pararas et al., 2011). The reciprocating flow system was later optimized to incorporate all of the fluidic components into a single, compact microfluidic device, where a digital control system was also introduced to manipulate the drug dosing pattern (e.g., switching between drug and water delivery) in guinea pigs to provide steady drug infusion (Tandon et al., 2016).

### Discussion

In general, pharmacotherapy has found its application in managing a wide array of non-genetic SNHL, ranging from those caused by acoustic trauma or ototoxic drugs (e.g., cisplatin, aminoglycoside antibiotics), to idiopathic SSNHL, as well as MD symptoms. The treatment commonly involved the administration of corticosteroids, such as dexamethasone and prednisolone.

In clinical use, corticosteroids were usually delivered systemically with i.v. injection or locally with intra-tympanic injection. Intra-tympanic delivery is believed to help the drug bypass the BLB and reach the inner ear at high concentrations. However, based on clinical results, there is no clear consensus on whether locally delivered corticosteroid provides higher therapeutic efficacy compared to systemic administration. Intra-cochlear administration of corticosteroid is also feasible but limited to perioperative use for reducing the inflammation associated with the surgical placement of cochlear implants.

In preclinical *in vitro* and *in vivo* studies, the formulation of corticosteroid has been engineered using a number of approaches, including hydrogels, nanocarriers, or a combination of both, to prolong the drug release and achieve higher drug concentrations in the inner ear. Drug delivery through mechanical devices, such as cochlear implant, osmotic pumps, a combination of both, or reciprocating perfusion system, provide continuous and direct infusion into the cochlea.

Future improvements in SNHL pharmacotherapy could potentially benefit from more in-depth understanding of the pharmacodynamics behind corticosteroids and otoprotectants, and how their mechanisms may vary for different etiologies in SNHL. Furthermore, novel biochemical or biomechanical designs for the drug delivery vehicles are required to meet the various needs, such as, targeted delivery with more precision, minimal invasiveness, and long-term drug elution.

## Gene Therapy and Delivery Methods

Sensorineural hearing loss can be caused by genetic mutations (Willems and Epstein, 2000), exposure to ototoxins (Bisht and Bist, 2011), noise trauma (Daniel, 2007), autoimmune disease (Mijovic et al., 2013), and aging (Gordon-Salant, 2005). Depending on the underlying pathology, genetic and non-genetic SNHLs may warrant different treatment strategies. Recently, gene therapy has been explored extensively in hope of expanding the toolbox for treating SNHL. In preclinical settings, gene replacement and gene interference have corrected genetic SNHL with known mutations at the RNA level. Gene editing enabled by clustered regularly interspaced short palindromic repeats (CRISPR) has corrected deafness-causing genes in DNA sequences *in vivo*. In addition to treating genetic SNHL, gene therapy has been used as a means for regenerative therapy to promote endogenous cell regeneration to restore hearing in non-genetic SNHL models, such as animals deafened by acoustic overexposure or ototoxins.

The cargoes of gene therapy may involve small molecule compounds (e.g., signaling pathway inhibitors), nucleic acid compounds (e.g., DNA, RNA, oligonucleotides), and proteins (e.g., enzymes, growth factors). Nucleic acids and proteins often require additional packing to prevent degradation in extracellular and intracellular environments. Viral vectors have been considered an efficient delivery vehicle for therapeutic transgene delivery to the cochlea. Common viral vectors used for the inner ear include Adenovirus (AdV), adeno-associated virus (AAV), Sendai virus, herpes simplex virus (HSV), and Lentivirus (Kanzaki, 2018; Kim et al., 2019). Specific viral vector is selected based on the target in the inner ear, which can include sensory cells (IHCs and OHCs), SGNs, supporting cells, and epithelial cells.

Adenoviruses are commonly used for transfecting supporting cells, but they do not enter sensory cells with high efficiency (Ishimoto et al., 2002; Kanzaki et al., 2002; Konishi et al., 2008). Meanwhile, AAVs show tissue tropism to sensory cells, but mostly in IHCs and not OHCs, due to reasons not fully elucidated yet (Kim et al., 2019). The small size of AAVs allow them to penetrate tissue barriers more easily but they are also restricted by a small carrier capacity of ∼4.7 kb. Genes larger than 4.7 kb require dual or triple AAV systems to reconstitute the transgene in target cells (Tornabene and Trapani, 2020). There are mixed reports on which AAV serotype has the highest transduction efficiency in the inner ear (Stone et al., 2005; Konishi et al., 2008; Askew et al., 2015). To date, the tropism of most viral vectors is not fully understood, but suggested to be partly related to the surface receptors used for viral entry, some of which are expressed in hair cells and supporting cells, for example, terminal galactose used by AAV9, heparan sulfate proteoglycan used by AAV2, sialic acid used by AAV5 (Maguire and Corey, 2020).

Another essential element for viral vector is an appropriate promoter region for the initiation of the transgene transcription. Cytomegalovirus (CMV) promotor is one of the most commonly used promoters for cochlear gene transfer because it can be efficiently activated in the hair cells, supporting cells, and SGNs in mouse models (Maguire and Corey, 2020). Chicken β-actin (CBA) promoter has strong activation activities in the hair cells and supporting cells in mouse models (Gu et al., 2019). If ubiquitous transgene expression in the inner ear is not desired, the glial fibrillary acidic protein (GFAP) and brain lipid-binding protein (BLBP) promoters which drive supporting cells-specific gene expression (in guinea pig models) can be used (Luebke et al., 2009).

Viral vectors could induce host-mounted immune reactions and carry the risk of integration of plasmid sequence into host genome. To overcome these issues, many non-viral carriers with lower immunogenicity have been developed, including lipid-based nanocarriers, dendrimers, polymersomes and inorganic nanoparticles (Young et al., 2016). These nanocarriers can be harnessed to deliver all of the aforementioned therapeutic cargoes and are sometimes preferred for delivering CRISPR-associated proteins (Cas) to reduce off-target editing (Glass et al., 2018), which will be discussed in more detail in the Subsection: Gene Editing.

These therapeutic agents are most frequently delivered through intra-cochlear or intra-labyrinthine injections. Cochleostomy allows direct access to the endolymph and improved transduction efficiency to the sensory cells but the surgery itself can also cause extensive damage to OHCs, leading to auxiliary hearing loss (Kilpatrick et al., 2011; Shu et al., 2016). In one study, the technical difficulty associated with apical cochleostomy induced tissue damage and resulted in less success in hearing rescue (5/30 mice) compared to RWM delivery of AAV vectors (19/19 mice) (Akil et al., 2012). Intra-labyrinthine surgeries, such as canalostomy (i.e., injection into the semicircular canal) and utricle injection, are typically less traumatic compared to cochleostomy because the injection site is located further away from the cochlea (Guo et al., 2018a; Lee et al., 2020). Both approaches have enabled AAV-mediated gene expression in cochlear hair cells and supporting cells in mouse models (Suzuki et al., 2017; Lee et al., 2020).

As a safer alternative to the surgeries, intra-tympanic transgene delivery has also been reported in a few studies. For example, cationic liposomes and AdVs placed outside the RWM onto a gelatin scaffold (Gelfoam) supported green fluorescent protein (GFP) transgene expression in nearly all tissue types in mouse cochlea with a base-to-apex gradient (Jero et al., 2001). Cell-penetrating peptides (CPPs) which are a class of short, cationic peptides (<30 amino acids) that can penetrate cellular membrane, have also been used for intra-tympanic delivery. An X-linked inhibitor of apoptosis protein (XIAP) has been directly modified with a CPP consisting of nine arginine groups. The purified XIAP-CPP was placed onto a gelatin sponge outside the RWM of guinea pigs prior to acoustic trauma and helped preserve OHC populations (*p* < 0.05 vs. untreated ear at 32 kHz) and partially rescued ABR threshold at 32 kHz (*p* < 0.05 vs. untreated ear) (Takeda et al., 2016).

### Gene Replenishment or Interference

There is a wide array of deafness-associated genes discovered in almost every cell type in the inner ear (Nishio et al., 2015). Several genetic defects, such as mutations on *Otof*, *Tmc1*, *Myo7A*, and *Cdh23* genes are known to interfere with hair cell stereocilia morphogenesis, mechano-transduction and ion channel transports; mutations affecting non-sensory cells may disrupt gap junction or other connective tissue networks in the inner ear, e.g., *Gjb2*, *Gjb6*, and *Cldn14* mutations; mutations affecting the extracellular matrix components of the tectorial membrane or the stria vascularis could also lead to deafness (Petit et al., 2001; Müller and Barr-Gillespie, 2015). The incidence of each of these mutations is rare, except for mutations affecting *Gjb2* which account for about 50% of all genetic SNHLs (Müller and Barr-Gillespie, 2015). Currently, efforts are focused on targeting mutations which affect the hair cells and supporting cells.

#### Transgene Delivery

Transgene delivery for replenishing copies of functional genes into the cochlea can be harnessed to treat autosomal recessive SNHLs which are typically caused by loss-of-function mutations and, to a lesser extent, autosomal dominant SNHLs involving gain-of-function mutations.

A transgene encoding the vesicular glutamate transporter-3 (VGLUT3), which is involved in hair cell glutamate release for synapses, has been delivered via AAV capsids in a knockout mouse model (Akil et al., 2012). Plasmid vectors delivered to P10–P12 mice restored VGULT3 expression in 40% of IHCs and repaired auditory synaptic transmission. ABR thresholds were near normal within 2 weeks after treatment (click stimulus and at 8–32 kHz) and remained within 10 dB of normalized range for 7 weeks.

**FIGURE 4:**
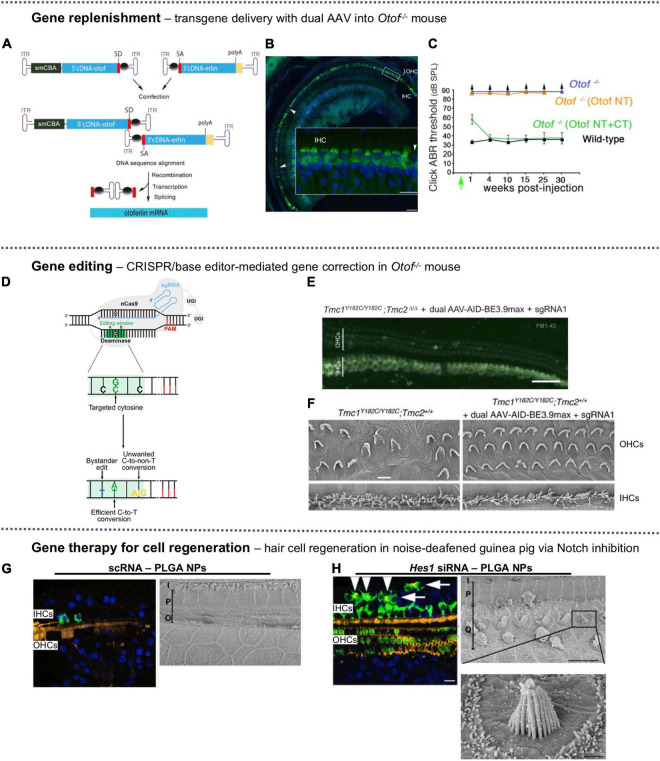
A selection of SNHL gene therapies studied *in vivo*. **(A)** Dual AAV-based packaging of the gene encoding Otoferlin, bridged by inverted terminal repeats (ITR). **(B)** The mid-to-apical turn of injected mouse cochlea showed strong expression of otoferlin (green) in inner hair cells (IHCs) but not in outer hair cells (OHCs), nuclear backstained in blue. **(C)** Auditory brainstem response (ABR) for dual-AAV injected mice (green) was similar to wild-type (black), but single-AAV injected mice (orange) and untreated mice (blue) had no measurable ABR threshold. The recombinant AAV-Otof NT and AAV-Otof CT vectors contain the 5′ and 3′ parts of the otoferlin cDNA, respectively. **(A–C)** Reproduced from Akil et al. (2019), available under Creative Commons license (CC BY-NC-ND 4.0). **(D)** A cytosine base editor composed of a nickase Cas9 (nCas9) fused to a deaminase can convert C:G base pair to T:A along with bystander edits or unwanted edits. Reproduced from Antoniou et al. (2021), available under Creative Commons license (CC BY 4.0). **(E)** Confocal images of mid-turn cochlea excised from base editing-treated Baringo mouse showing uptake of FM1-43 (green) in IHCs and OHCs, indicating restored mechanotransduction (scale bar = 50 μm). **(F)** Scanning electron microscopy (SEM) images of apical OHCs and IHCs of [left] untreated Baringo mouse and [right] base editing-treated Baringo mice (scale bar = 10 μm). **(E,F)** Reproduced from Yeh et al. (2020) with permission. **(G)** Sham-treated (scRNA) noise-deafened guinea pig [left] mid-turn hair cells immunostained with anti-myosin VIIa (green), stereocilia with phalloidin (yellow), nuclei (blue) (scale bar = 50 μm) and [right] SEM showing complete ablation of basal OHCs (scale bar = 10 μm). **(H)** Noise-deafened guinea pig treated with *Hes1* silencing RNA (siRNA) for Notch inhibition [left] immunohistochemical staining showing supernumery IHCs; arrowheads indicate ectopic IHCs with stereocilia, arrows indicated those without (scale bar = 50 μm) and [right] SEM showing regenerated basal OHCs, some with abnormal stereocilia lacking the canonical stair-step organization (scale bars = 10 μm and 1 μm). **(G,H)** Reproduced from Du et al. (2018), available under (CC BY-NC-ND 4.0).

A gene encoding the otoferlin protein involved in Ca^2+^-triggered sensory synapse, *Otof* (∼6 kb), is large enough to entail a dual AAV system (Akil et al., 2019). Two AAV2 vectors, each carrying one half of the murine otoferlin complementary DNA (cDNA) modified with a recombinogenic bridging sequence (inverted terminal repeats) (Figure 4A), have been delivered across the RWM of profoundly deaf *Otof^–/–^* mice at P10. Otoferlin expression was detected in 64% of the IHCs of treated animals. The ABR thresholds (at 8, 16, 32 kHz) were restored to near-normal levels and maintained for 30 weeks post-treatment (Figures 4B,C). The same treatment applied to P19 and P30 *Otof ^–/–^* mice resulted in similar transgene expressions in the IHCs at 82 and 85%, respectively, and near-normal ABR threshold.

In an Usher syndrome type IIIA (USH3A) mouse model, where *Clrn1* mutation adversely affects the stereocilia morphogenesis and synapse of hair cells, recombinant AAV 2/8 (denoting a hybrid vector where a recombinant AAV2 genome is packed into an AAV8 capsid) carrying clarin-1 cDNA was injected via the RWM at P2–P3. The treatment demonstrated 90% transduction efficiency in IHCs and 20% in OHCs (Dulon et al., 2018). This gene therapy corrected stereocilia morphogenesis and preserved the hearing sensitivity of conditional *Clrn1* knockout animals (ABR threshold = 20 dB at 10 kHz) compared to the untreated group (ABR threshold = 67 dB at 10 kHz).

Transmembrane channel-like (TMC) proteins TMC1 and TMC2 are involved in stereocilia mechanotransduction machinery. Mutations on TMC1 comprise about 3% of genetic hearing loss in human, including the autosomal recessive deafness DFNB7/11 and the autosomal dominant DFNA36 (Sloan-Heggen et al., 2016). *Trans*-RWM injection of AAVs has been explored to introduce *Tmc1* and *Tmc2* genes into TMC1-knockout mice (*Tmc1*^Δ^*^/^*^Δ^) and Beethoven (*Bth*) mice at P0–P2 (Askew et al., 2015). AAV2/1-*Tmc1* vectors injected into *Tmc1*^Δ^*^/^*^Δ^ mice (model for DFNB7/11) induced Tmc1 protein expression in the IHCs (∼65%) and the OHCs (∼5%) with base-to-apex gradient, and showed improvements in ABR response (85–100 dB at 5–16 kHz) compared to untreated group (>115 dB at 5–32 kHz). However, the DPOAEs of treated animals were similar to the untreated group, indicating little recovery of OHC function. Using the same vector construct, AAV2/1-*Tmc2* injected into *Bth* mice (model for DFNA36) resulted in a similar partial recovery of response of 90–110 dB at 5–16 kHz compared to untreated *Bth* mice (>115 dB at 5–32 kHz) (Askew et al., 2015).

Adeno-associated virus vectors were also used to deliver *Gjb2*, a gene encoding the gap junction beta-2 protein (GJB2, also known as connexin 26) in the sensory cells of *Gjb2*-deficient mice (Iizuka et al., 2015). RWM injection of AAV vectors into neonatal P0 mice restored the formation of the tunnel of Corti, preserved SGNs, and lowered ABR threshold by 20–30 dB at 12 and 24 kHz, compared to untreated ear. Again, the restored morphology was more evident in the basal turn compared to the apical turn, hinting at the effect of slow diffusion or tissue tropism of AAVs. This treatment did not restore hearing in adult *Gjb2*-deficient mice, however, although GJB2 expression was detected, revealing a common limitation of SNHL gene therapies concerning the narrow treatment window for restoring normal auditory organ development (Iizuka et al., 2015).

Many AAV-mediated gene therapies for treating SNHL were conducted in neonatal mice. This was partly due to AAV uptake in hair cells becoming less effective as neonatal mouse mature (Akil et al., 2012; Shu et al., 2016; Suzuki et al., 2017; György et al., 2019a; Lee et al., 2020) and also because mouse cochlea continues to develop until P15 (Kopecky et al., 2012). When the same treatment was carried out in non-neonatal mice, the therapeutic effects usually diminishes. For example, AAV1-mediated injection of *Kcne1* gene into Jervell and Lange-Nielsen syndrome type 2 (JLNS2) mice at P3 failed to preserve hearing while injections performed at P0–P2 preserved auditory function in 80% of mice (Wu et al., 2021).

Another complication for gene replacement therapy is that hearing loss rescue may not be sustained for the lifetime as the introduced exogenous gene tend to diminish over time (Iizuka et al., 2015) while protein recycling is a continuous process (Schneider et al., 2002). In one instance, *Myo15a* transgene introduced to mouse zygote was able to correct inner ear structure and function for up to 6 months before partial hearing loss started to recur (Kanzaki et al., 2006). AAV-mediated delivery of the gene encoding VGLUT3, after restoring near normal ABR threshold in 100% of mice, witnessed decline in auditory rescue after 7 weeks (Akil et al., 2012).

#### Gene Silencing

Around 20% of genetic SNHL in human are caused by gain-of-function mutations (Yeh et al., 2020), the treatment of which could be accomplished through silencing the expression of mutated allele or through the aforementioned transgene delivery to increase expression of the correct copies of the gene. Therapeutic application of RNA interference is achieved through the introduction of synthetic, short non-coding RNA (20–30 nucleotides), among which small interfering RNAs (siRNAs) and microRNAs (miRNAs) have attracted considerable interest (Carthew and Sontheimer, 2009). The siRNAs are double-stranded and highly specific in targeting a gene of interest, while the single-stranded miRNAs could have multiple gene targets, but both siRNAs and miRNAs can activate the formation of RNA-induced silencing complexes (RISCs) to prevent messenger RNA (mRNA) translation to achieve gene silencing (Lam et al., 2015).

A single intracochlear injection of artificial miRNAs enclosed in AAV2/9 vectors in *Bth* mouse on P0–P2 suppressed the expression of a semi-dominant point mutation on *Tmc1* (c.1235T > A) (Shibata et al., 2016). Levels of mRNA expression of the mutant allele was suppressed by >88% compared to untreated ear. Consequently, hearing loss was slowed for approximately 21 weeks, at which point click ABR threshold shifted by >20 dB compared to wild-type control. The AAV2/9 showed 74% transduction efficiency in IHCs at the apical turn but only 7% efficiency in the OHCs. Consequently, IHC cell count was significantly improved compared to untreated ear at the apical turn (*p* < 0.005) but OHC survival showed no improvement.

Small interfering RNAs (siRNAs) were also tested in mice which were infected with a transgene carrying a dominant *Gjb2* mutant allele (Maeda et al., 2005). The siRNAs were complexed with liposomes and placed against the RWM of adult mice. Expression of the exogenous mutant *Gjb* allele was suppressed by over 70% while endogenous *Gjb* expression was unaffected. Click ABR threshold was recovered to similar level as control animals (14.5 dB higher than control) although infected animals were not exposed to a severe shift in ABR threshold in the first place (23 dB higher than control).

Small antisense oligonucleotides (ASOs) have been used to correct Usher syndrome type 1C (USH1C) in a mouse model, by redirecting the splicing of the mutant *Ush1c* allele (216G > A) (Lentz et al., 2013). The ASOs were dissolved in saline and injected intraperitoneally at a dose of 50 mg/kg – 300 mg/kg into P3–P5 mice. The ASOs partially rectified the pre-mRNA splicing of *Ush1c*, enabled the production of harmonin which is involved in stereocilia morphogenesis and restored cochlear hair cell morphology. ABR response at lower frequencies (8, 16 kHz) were similar to those of normal ears but ABR threshold at 32 kHz was similar to untreated ears, suggesting high-frequency hearing was not rescued.

Together, these findings demonstrate that various gene silencing approaches can be harnessed *in vivo*, to selectively inhibit the expression of the mutant alleles that lead to dominant gain-of-function mutations. These therapies almost always require packaging vehicles to protect the genes or small oligonucleotides from degradation catalyzed in part by nucleases and lysozymes, but these vehicles may also elicit varying degrees of immunological response, especially viral vectors which can initiate both adaptive and innate immune responses (Kanasty et al., 2012; Ronzitti et al., 2020). Gene silencing therapies may also be limited by potential innate immune responses mounted against the double-stranded siRNAs, resulting in increased secretion of interferons and proinflammatory cytokines, which may be detrimental to the inner ear (Meng and Lu, 2017). Such immune responses can also be modulated, or nearly abrogated by careful design of siRNAs, such as, avoiding the use of uridine and guanosine motif or the use of synthetic nucleoside replacements (Judge et al., 2005, 2006; Sajid et al., 2020).

#### The Challenge of Cell-Specific Adeno-Associated Virus Delivery and the Development of Synthetic Adeno-Associated Virus Capsids

It has been a recognized challenge that *in vivo* AAV transduction in the inner ear is mostly limited to IHCs, even when experiments with *ex vivo* cochlear explants show similar transduction efficiencies between IHCs and OHCs. Efforts to improve viral vector uptake in the OHCs include (1) creating synthetic AAV capsids with higher OHC-targeting abilities and (2) exploring different intra-cochlear or intra-labyrinthine injection methods.

Several synthetic AAV variant capsids with differed or selective cell-targeting abilities have been tested. For example, AAV2/Anc80L065, a synthetic vector which approximates the common ancestor of AAV serotypes 1, 2, 6, 8, and 9, was tested to infect IHCs and OHCs and showed better transduction efficacy compared to conventional AAV serotypes in neonatal mice following RWM injection (100% IHCs and ∼90% OHCs, compared to <5% OHCs in natural serotypes) (Landegger et al., 2017). The synthetic capsid AAV/Anc80L065 was later used to deliver wild-type *Ush1c* into a mouse model (*Ush1c c.216G* > *A)* through RWM on P0–P1. Plasmid injection induced a significant recovery of mechanosensitivity in both OHCs and IHCs (*p* < 0.001) and a prominent recovery of ABR threshold at lower frequencies (25–30 dB at 5.6–16 kHz). However, when injected on P10–P12, the treatment did not provide therapeutic effects (Pan et al., 2017). AAV9-PHP.B, a synthetic vector first engineered for transport across the BBB (Deverman et al., 2016), has also shown high transduction efficacy in IHCs (50% - 70%) and OHCs (30–40%) via RWM injection in neonatal mice, but did not transfect supporting cells in the sensory epithelium (György et al., 2019a). The synthetic vector AAV2.7m8 was identified from an *in vivo* directed evolution screening for mouse retinal gene delivery (Dalkara et al., 2013). When applied to cochlear gene delivery, AAV2.7m8 loaded with GFP was shown to infect IHCs and OHCs in mice, in addition to the inner pillar cells and inner phalangeal cells. AAV2.7m8 has been shown to infect murine OHCs at higher efficiency (83%) than AAV/Anc80L65 (67%) when injected via canalostomy into mice (Isgrig et al., 2019). The three aforementioned synthetic vectors, i.e., AAV2/Anc80L5, AAV9-PHP.B, and AAV2.7m8, have been compared in mice cochleae, where AAV9-PHP.B showed the highest transduction efficiency, at nearly 100%, for both IHCs and OHCs following utricle injection (Lee et al., 2020).

The exact route of injection may also influence AAV uptake in the organ of Corti. AAV delivered via trans-RWM injection into the perilymph were largely undetectable in the OHCs (Stone et al., 2005; Konishi et al., 2008; Dulon et al., 2018; Kim et al., 2019). In other studies where AAVs were delivered directly into the mouse endolymph via cochleostomy (Kilpatrick et al., 2011) or canalostomy (Tao et al., 2018), uptake for a few AAV serotypes (e.g., AAV2, AAV8) was observed in the OHCs (0–20%), but still at a much lower incidence rate compared to IHCs (40–100%). Utricle injection, which also directly access the endolymph, of a synthetic vector AAV9-PHP.B in mice supported a higher OHC transduction rate (100%), compared to that observed after RWM injection (40–70%) (Lee et al., 2020), suggesting that endolymphic delivery may present an advantage over perilymphic delivery for the purpose of transducing sensory cells.

### Gene Editing

Discovery of the CRISPR system in prokaryotes and its repurposing into a powerful gene editing tool in mammalian cells have introduced a new weaponry for gene therapy. One of the most common designs for introducing permanent genome editing comprises three components: (i) the Cas9 protein, i.e., an RNA-guided DNA endonuclease, (ii) a single guide RNA (sgRNA) for mapping to the target DNA region, and (iii) if homologous recombination is desired, a single-stranded oligodeoxynucleotide (ssODN) to serve as the donor template (Anzalone et al., 2020). After Cas9 cleaves the DNA at the targeted site, the double strand breaks can be re-ligated through a few repair mechanisms, including (1) the error-prone non-homologous end joining (NHEJ), which is the major repair pathway and is active during all stages of the cell cycle, and (2) the high-fidelity homology directed repair (HDR), which requires a DNA repair template and is active only in the late S or G2 phase as cells prepare for mitosis (Anzalone et al., 2020). Base editing and prime editing have also recently been added to the CRISPR toolbox (Kantor et al., 2020).

In the context of hearing loss treatments, Cas9 was first used to correct the mutations in induced pluripotent stem cells (iPSCs) *ex vivo* using cells derived from SNHL patients. Mutations affecting *Myo7a* in DFNB2 and DFNA11 deafness, and *Myo15a* in DFNB3 deafness have been genetically corrected through HDR. The hair cell-like cells derived from the edited iPSCs showed morphology and function reminiscent of native hair cells (Chen et al., 2016; Tang et al., 2016). After confirming the success of *ex vivo* edits, a small number of proof-of-concept *in utero* or *in vivo* gene editing therapies have been explored, which are detailed in the next few subsections, followed by a discussion on the delivery vehicle for CRISPR-editing machineries.

#### Cas9 Non-homologous End Joining-Mediated Gene Disruption

Non-homologous end joining generates multiple random nucleotide insertion or deletions (indel) at the repair junction (e.g., in a mouse embryonic stem cell line, indels can account for ∼50% of all NHEJ events) (Guo et al., 2018b) which can disrupt the open reading frame, therefore NHEJ can be harnessed to silence dominant alleles involved in hearing loss, which accounts for about 20% of all genetic deafness (Angeli et al., 2012).

The use of Cas9-sgRNA complex to disrupt the dominant point mutation on *Tmc1* (p.M418K, c.T1235A) has been demonstrated in *Bth* mouse (a model for DFNA36 hearing loss) (Gao et al., 2017). In this study, Cas9 and sgRNA were delivered as a ribonucleoprotein (RNP) complex using Lipofectamine 2000, a cationic liposome formulation, via cochleostomy into the scala media of *Tmc1*^Bth/+^* Tmc2^+/+^* mice on P1. Allele-specific disruption occurred in up to 10% of sampled organ of Corti cells from treated mice. IHC and OHC survival was enhanced by ∼80% and ∼30%, respectively, at location corresponding to 32 kHz and stereocilia bundles were preserved at 16 and 32 kHz whereas untreated ear suffered almost complete loss of stereocilia at these locations. In terms of hearing function, the treated mice showed reduction in ABR threshold by ∼15 dB compared to untreated mice, with the difference being most evident at lower frequency ranges (8–23 kHz). The treated ears also demonstrated slight change in DPOAE (∼20 dB shift at 16 kHz), suggesting possible OHC damage from the surgical procedure of cochleostomy.

Combinations of Cas9 variants and optimized sgRNAs have later been investigated to improve the efficiency of Cas9-mediated *Tmc1* gene disruption in *Bth* mouse (György et al., 2019b). In this study, AAV/Anc80L65 with CMV promoter was used and the exact route of intra-cochlear injection was not disclosed. When using AAV vectors instead of liposomes, the Cas9-Tmc1 sgRNA combination used in the previous study (Gao et al., 2017) lost the reported allele specificity when editing *Bth* mouse fibroblasts, suggesting perhaps an unresolved influence of the route and format of Cas9 delivery. Thereafter, the authors selected a Cas9 variant which recognizes a specific protospacer-adjacent motif (PAM) site that is present in the mutant allele of *Tmc1* but not in the wild-type allele. In heterozygous *Bth* mouse fibroblasts, 98% of all indels on *Tmc1* occurred preferentially in the mutant allele, mostly as a frameshift mutation which disrupted the gene. When injected into *Bth* mice on P1, this Cas9 variant lowered the expression of *Bth* mRNA in cochlear tissue by 24%. ABR threshold improved by about 20 dB compared to the untreated group (at 8–22 kHz) while DPOAE showed near-normal function of OHCs (at 5–11 kHz).

#### Cas9 Homology Directed Repair-Mediated Gene Correction

Homology directed repair-mediated high-fidelity repair can be harnessed to correct recessive, loss-of-function mutations, which are responsible for the remaining ∼80% of genetic deafness (Yeh et al., 2020). In addition to native Cas9 enzymes, Cas9 nickase (a mutated Cas9 which only creates single-strand DNA nick) has been adopted since DNA nicks can be repaired with HDR with higher fidelity compared to the toxic double-strand breaks and incur lower off-target edits.

The HDR pathway was harnessed to repair the *Cdh23^ahl^* allele in a C57BL/6NTac mouse model, which mimics the progressive hearing loss DFNB12 and USH1D syndrome in human caused by a single nucleotide substitution on *Cdh23^ahl^* (c.753) (Mianné et al., 2016). In this study, a Cas9 nickase (D10A) with a pair of sgRNAs was used to produce two single strand breaks with a 5′ overhang, reducing off-target activities from double-strand producing-Cas9 enzymes. After the DNA cleavage, a 121 bp ssODN was used as the donor template for homologous recombination to repair the *Cdh23* gene. The Cas9 nickase mRNAs, sgRNAs, and ssODNs were injected into one-cell state mouse embryos. Of the 456 embryos injected, 104 gave rise to live pups (22.8%), of which 15 were transgenic at the target site (14.4%). Four pups carried the correct *Cdh23*^753*A*>*G*^ repair (3.8%) while the other 11 pups carried incorrect repairs from HDR. Heterozygously repaired littermates (*Cdh23*^*ahl/*753*A*>*^G^*^) showed normal IHC and OHC morphology throughout the cochlea, whereas untreated animals suffered from >50% OHC loss at the basal turn (hair cell morphology was normal at other regions). ABR threshold was reduced by >25 dB at 32 kHz in the treated group. ABR response at 8 and 16 kHz were within normal range for both treated and untreated group.

#### Base Editor-Mediated Gene Correction

A base editor is a fusion protein comprised of a Cas9 nickase or a catalytically inactive ‘dead’ Cas9 (dCas9) fused onto a deaminase. Base editors can directly exchange one base pair for another at the target locus, relying on the cellular mismatch repair machinery instead of HDR, and therefore is not limited only to mitotic cells (Komor et al., 2016; Kantor et al., 2020). Prime editors further expand the scope of donor-free DNA editing to cover all possible point mutations and small insertion or deletion. Prime editors comprise a Cas9 nickase fused to a reverse transcriptase, which uses a long, single-strand prime editing guide RNA (pegRNA) to perform the desired sequence change, and are also well tolerated in post-mitotic cells (Anzalone et al., 2019; Kantor et al., 2020).

The use of base editing to repair deafness-causing point mutations was first demonstrated in a Baringo mouse model (*Tmc1^*Y*182*C/Y*182*C*^*; *Tmc2*^+/+^), which harbors a recessive, loss-of-function mutation on *Tmc1* (*c.A545G*, p.Y182C) that mimics the congenital hearing loss DFNB7/B11 in human (Yeh et al., 2020). The selected cytosine base editor (∼5.2 kb) (Figure 4D) exceeded the maximum loading capacity of a single AAV (∼4.7), and therefore was slightly truncated and split in half to be encapsulated in a split-intein, dual-AAV system. AAV/Anc80L65 with Cbh promoter was injected into the inner ear of P1 Baringo mouse. The exact route of injection was not disclosed. On-target base editing in sampled cochlear cells occurred at an efficiency of 10 - 51% and off-target editing was minimal. Editing restored the normal IHC hair bundle morphology at the apical end and partially in the basal end (Figure 4F). Some treated mice (9/15) showed slight recovery of ABR response (5–50 dB improvement at 5.6 kHz) and the remaining 6/15 treated mice showed no detectable ABR response. DPOAE response was not seen in any treated mice, suggesting a lack of functional recovery of the OHCs. This might be in part due to the low transduction efficiency of Anc80L65 and the promoter, or a combination of both (expression of GFP was 22.6–41.7% in IHCs, and 2.6–8.3% in OHCs) (Figure 4E). Another study investigated the use of Anc80L65 with CMV promoter to deliver Cas9 into the cochlea via cochleostomy in P1-P3 mice or canalostomy in adult mice also showed that Anc80L65 had almost no transduction in the OHCs although IHCs were completely infected (Kang et al., 2020).

#### Delivery Vehicles for Gene Editors

Cas9 can be delivered in the form of DNA plasmid, mRNA, or protein. Most of the aforementioned transgene delivery strategies, especially those deployed *in vivo*, also apply to the delivery of Cas9 systems in plasmid forms. Ultimately, for editing to take place, the RNP of Cas9-sgRNA must be present inside the cell and translocate into the cell nucleus. AAV vectors are commonly used for assisting Cas9 *in vivo* in bypassing cellular barriers and evading cellular degradation pathways. One limitation of AAV delivery is its loading capacity (∼4.7 kb). The restricted cargo size poses difficulties for packaging the Cas9 (e.g., the commonly used *Streptococcus pyogenes* Cas9 is about 4.2 kb) and sgRNA into a single DNA plasmid. As a solution, smaller Cas9 orthologs (e.g., the *Staphylococcus aureus* Cas9 which is about 3.2 kb) or separate plasmids for Cas9 and sgRNA may be used (Ran et al., 2015). Base editors and prime editors also exceed the loading capacity and therefore need dual-AAV systems. To promote nuclear transport, Simian vacuolating virus 40 (SV40) nuclear localization sequence (NLS) are often encoded into Cas9 DNA or RNA constructs or attached to the terminal of Cas9 proteins (Staahl et al., 2017).

An important consideration when designing a delivery system for Cas9-based therapy is that, different from other gene therapy reagents, the long-term expression of Cas9 may not be desirable. A transient, one-time expression of Cas9 in the host cell can shorten the time window of editing and reduce off-target edits. Delivery of Cas9 and sgRNA as an RNP complex is therefore appealing due to the fast onset of the gene editing machinery and the non-replicable nature of protein agents. Comparing the gene modification specificity of Cas9-sgRNA delivered as viral plasmid vs. RNP in HEK293T cells, cationic-lipid mediated RNP delivery showed a 19-fold higher on-target/off-target editing rate compared to plasmid delivery and the on-target modification rate (10%) was comparable between the two methods (Zuris et al., 2014). Compared to viral plasmids, liposomes or other nanoparticle-based strategies are also favorable because they avoid the risk of integration of the plasmid sequence into host genome and thus likely lowers host-mounted immune reactions against Cas9 entry.

Although native Cas9 protein carries positive charge and is therefore unlikely to be encapsulated into cationic lipids, it can be combined with a sgRNA, which is rich in phosphate groups and carries multiple negative charge, to become compatible with cationic liposomes (Zuris et al., 2014). The liposomes can then interact with the negatively charged cell surface, carrying the RNP into the cell. Moreover, liposomes may facilitate endosomal escape, increasing the likelihood that RNPs can reach the nucleus for access to the transcriptional machinery (Dalby et al., 2004). As with how different virus serotypes show differed transfection efficiency, various commercialized cationic lipid transfection kits also show variable degree of efficacy. In one study, Lipofectamine 2000 showed higher efficacy (12% indel) than RNAiMAX (7.7%) or CRISPRMAX (8.9%) for delivering the Cas9-Tmc1 RNP of interest (Gao et al., 2017). Liposomes can also be used to deliver Cas9 in the form of DNA plasmids and mRNA strands.

Lipoplex has also been used to deliver Cas9 RNP. A cationic cell-penetrating peptides (CPP) containing 9 Arginine groups was used to create two groups of cationic nanoparticles from CPP-conjugated Cas9 proteins and CPP-complexed sgRNAs (Ramakrishna et al., 2014). These NPs effectively transfected HEK293T cells *in vitro*, but have not been tested *in vivo*. Inorganic NPs represent another strategy for Cas9 RNP delivery. A CRISPR-Gold complex was designed with a gold NP core conjugated to a layer of DNA, which was then complexed with ssODN and the Cas9 RNP. This complexation was then covered with a layer of cationic endosomal disruptive polymer, poly(*N*-(*N*-(2-aminoethyl)-2-aminoethyl) aspartamide) [PAsp(DET)] (Lee et al., 2017). CRISPR-Gold showed success in enabling HDR gene editing in mice through non-clathrin-mediated endocytosis, but has not been used for treating hearing loss yet. In another design, the negatively charged Cas9 RNPs self-assembled with cationic arginine gold nanoparticles to form nanoassemblies which can enter HeLa cells through a membrane fusion process instead of endocytosis and demonstrated ∼30% indel efficiency *in vitro* (Mout et al., 2017).

### Gene Therapy for Cell Regeneration

Unlike genetic SNHL, non-genetic SNHL patients commonly have normal cochlear development but later suffer from a deafness-causing incidence. Unfortunately, damage to the cochlear sensory cells is generally irreversible in human (Cheng, 2017). Mammalian organ of Corti remains in a quiescent state throughout adult life and possesses little plasticity and very limited regenerative potential (Oshima et al., 2006; Hartman et al., 2009). As the number of hair cells decreases, synaptic signals stimulating the SGNs also decrease, leading to permanent neuron degeneration. The supporting cells also rapidly degenerate after the death of hair cells until the epithelium in the organ of Corti become flattened. Regeneration of hair cells and SGNs could ideally fundamentally repair cochlear function to restore hearing.

#### Hair Cell Regeneration

Hair cells have been hypothesized to regenerate from two pathways: (i) transdifferentiation of supporting cells without cell divisions, or (ii) mitotic regeneration from progenitor cells, which is more difficult to achieve (Shibata et al., 2020). Regenerative therapies targeting the inner ear often modulates the highly conserved Wnt and Notch signaling pathways, which are both critical for cell proliferation, differentiation, and fate determination in early morphogenesis (Samarajeewa et al., 2019). These two pathways have a complex interplay for hair cell formation (Zheng and Gao, 2000; Li et al., 2015; Cheng, 2017).

Notch signaling upregulates several effectors, including the hairy and enhancer of split-1 (Hes1) which downregulates the expression of basic helix-loop-helix transcription factors such as atonal homolog 1 (Atoh1) (Samarajeewa et al., 2019). Strategies to inhibit Notch signaling or directly upregulating Atoh1 have induced the formation of new cochlear hair cells (Woods et al., 2004; Bramhall et al., 2014). The canonical Wnt signaling pathway elevates the cytosolic concentration of β-catenin, which is an effector that can translocate to the nucleus and bind with T cell-specific factor/lymphoid enhancer-binding factor (TCF/LEF) and co-activators to initiate the transcription of target genes (Samarajeewa et al., 2019).

In the organ of Corti, a small group of supporting cells express G-protein-coupled receptor (Lgr5), and are believed to possess progenitor-like activities (Shi et al., 2013). In transgenic mice, the Lgr5-positive cells have been shown to undergo mitotic generation into hair cells following forced expression of β-catenin, a mediator of the canonical Wnt signaling pathway (Shi et al., 2013). The Lgr5-positive cells (i.e., inner pillar cells and the third row of Deiters cells) have also been shown to spontaneously transdifferentiate into hair cells after Notch inhibition in gentamicin-damaged newborn mouse cochlea (Bramhall et al., 2014).

Mounting evidence has suggested that Notch inhibition more likely promotes hair cell regeneration from supporting cell transdifferentiation rather than from mitotic regeneration (Li et al., 2015). In one study, the *Atoh1* gene was carried in AdV vectors and injected into the endolymph of young adult guinea pigs. Atoh1 expression was induced in the supporting cells; these immature hair cells formed mostly in the organ of Corti and some ectopic hair cells were found in adjacent areas with evidence of neurofilament extension to these cells (Kawamoto et al., 2003). In a later study, AdV-Atoh1 was delivered via cochleostomy into the scala media of young adult guinea pigs, after complete loss of hair cells (but preservation of supporting pillar cells) due to high dose of kanamycin. Atoh1 induced the formation of new hair cells with similar surface morphology as normal hair cells. An average of 256 IHCs and 691 OHCs were detected in a 2 mm organ of Corti region compared to no cells detected in untreated ear. Some of these new cells had mixed phenotype of OHC and supporting cells, suggesting that supporting cells have transdifferentiated into hair cells. A significant increase in the number of nuclei (1548 vs. 830 nuclei in untreated ear) also suggest mitotic events for hair cell regeneration. ABR recovery in Atoh1 inoculated ear was significantly improved compared to the untreated ear (4–24 kHz, *p* < 0.004) (Izumikawa et al., 2005).

In another study, overexpression of *Atoh1* was suggested to treat hearing loss via stabilizing injured hair cells and promoting stereocilia repair (Yang et al., 2012). In this guinea pig model of noise-induced hearing loss, injured hair cells and supporting cells were both present before the treatment of *Atoh1*-loaded AdV through trans-RWM injection and the repair of existing hair cell was purposed to be due to regeneration of damaged stereocilia instead of supporting cell transdifferentiation. Improvements in ABR threshold of 35–40 dB were noted at 4–20 kHz compared to untreated ear. When the same treatment was carried out at 1 month after noise exposure, none of the animals recovered hearing, supporting the notion that the presence of hair cells was necessary for the suggested *Atoh1*-induced repair.

Another Notch inhibition strategy involved using siRNAs (encapsulated in PLGA nanoparticles) directed against Hes1, a downstream effector of Notch signaling (Du et al., 2018). The formulation was delivered into the cochlea of young adult guinea pigs at 72 h after acoustic deafening, via a mini-osmotic pump placed through cochleostomy for 24 h infusion. New IHCs and OHCs were observed mostly at the basal and second turn of the cochlea which were proximal to the infusion site. SEM images of the organ of Corti at basal turns showed that OHCs were completely ablated in noise-injured cochlea (Figure 4G) whereas in PLGA-Hes1 treated cochlea, OHCs were present, although many were equipped with immature stereocilia showing shorter, incorrectly angled hair bundles (Figure 4H). ABR threshold recoveries at 3–9 weeks post-treatment exhibited a base-to-apex gradient, with more recovery witnessed at higher frequency (∼21 dB improvement at 16 kHz vs. ∼9 dB at 2 kHz) (Du et al., 2018).

Adenoviruses are popular among *Atoh1* transgene therapies for hair cell transdifferentiation, but AAV are much less commonly used because of their low transduction rate in supporting cells. An AAV variant, AAV-ie, was made by the insertion of a CPP-like peptide derived from a neuron-targeting vector, AAV9-PHP.eB (Chan et al., 2017), into the capsid of a liver-targeting and low-immunogenic vector, AAV-DJ (Grimm et al., 2008). The newly constructed AAV-ie was shown to infect a broad range of cells in the cochlea at high efficiencies, including IHCs, OHCs, supporting cells, and SGNs, following intra-RWM injection into mouse cochlea (Tan et al., 2019). At the apical turn, AAV-ie transduced 77% of supporting cells, while PHP.eB, AAV-DJ, and AAV/Anc80L65 transduced < 20%, <55%, and <55% of supporting cells, respectively. *Atoh1* delivered through AAV-ie vector via the RWM at P0 into normal C57BL/6 mice induced ectopic new hair cell formation in the sensory epithelium and the greater epithelial ridge. The new hair cells possessed hair bundles and some excitatory characteristics (Tan et al., 2019). However, due to the broad tropism of AAV-ie, it was difficult to analyze the exact action site of Atoh1 in this experiment, and this property of AAV-ie may limit its therapeutic applications, but may be solved by cell-specific promoters, such as GFAP which is only expressed in supporting cells but not hair cells.

#### Neuron Regeneration

Following the death of hair cells, spiral ganglion cells, which are the sole messengers in the cochlea for relaying auditory information from the hair cells to the central auditory pathway in the brain, also gradually degenerate as they cease to receive sensory signals and neurotrophic factors from the organ of Corti (Spoendlin, 1975; Fritzsch et al., 1997; Hardie and Shepherd, 1999). Degeneration of the SGN can limit the success of hair cell-targeted therapies or the outcome of cochlear implants which electrically stimulate any residual SGNs to restore hearing perceptions (Ahmed et al., 2017). Exogenous replenishment of several neurotrophins, including brain-derived neurotrophic factor (BDNF), glial cell line-derived neurotrophic factor (GDNF), and neurotrophin3 (NT3) have been used to rescue SGN after hair cell damage. For example, BDNF proteins delivered via a mini-osmotic pump (Alzet) for 28 days into chemically deafened rat cochleae (flow rate 0.25 μl/h) rescued SGN density to statistically similar level to that of normal rats while non-treated group suffered from significant SGN loss (*p* < 0.001) (McGuinness and Shepherd, 2005). Similarly, BDNF proteins delivered via osmotic pump for 28 days into chemically deafened guinea pig cochleae also showed preservation of all three elements of the SGNs (peripheral processes, somata, axon) and electrically evoked compound action potentials which were similar to that from normal hearing cochlea for the duration of BDNF infusion and at least 8 weeks after termination of treatment (Ramekers et al., 2015; Vink et al., 2021).

These neurotrophic factors can also be delivered as genetic material. GDNF transgene packed in AdV vectors were delivered to chemically deafened guinea pigs through the scala tympani 4 days after deafening. SGN cell survival was 10–30% higher in GDNF group compared to non-inoculated control ears (Yagi et al., 2000). Auditory function assessment was not reported. In another study, NT-3 cDNA packed in HSV-1 vector was inoculated into the scala vestibuli of murine cochlea. Expression of NT-3 lowered cisplatin-induced SGN apoptosis or necrosis and preserved SGN survival by more than 60% compared to control virus injection (Bowers et al., 2002).

In addition to viral vector-mediated packaging, neurotrophins were also successfully encapsulated in synthetic nanoparticles in the form of mRNA and proteins. Non-viral delivery bypasses the safety concern with exogenous viral particles and avoids the risk of DNA plasmid integration into host genome. As an example, nanoporous silica NPs was used to package BDNF proteins and demonstrated sustained release in fibroblast cell culture for up to 39 days and significantly improved SGN survival *in vitro* (*p* < 0.001) (Santos et al., 2018). A neutral lipid nanoparticle (LNP) was also developed for encapsulating BDNF mRNA (Miwa et al., 2020). The LNPs were placed onto a gelatin sponge outside the RWM of guinea pigs at day 1 or day 14 after gentamicin-induced hearing loss. Both day 1- and day 14-BDNF treatment rescued SGN survival to near normal level, but untreated groups suffered significant SGN cell loss (*p* < 0.001). OHC survival was similar between day 1-BDNF group and control but day 14-BDNF group suffered significant OHC loss (*p* < 0.01). ABR thresholds measured from day 1-BDNF group were similar to that of normal ear (4–32 kHz) but the day 14 BDNF-group showed ABR response similar to untreated group.

### Discussion

To summarize, recent advances in strategies for genetic manipulation revealed new possibilities for treating genetic SNHL. These strategies include replenishing functional copies of the target gene with transgene delivery, silencing mutant copies at the transcriptional or translational level, and using CRISPR-mediated editing to permanently correct or suppress the target gene in host genome. An intersection exists between gene therapy and regenerative therapy, which enables endogenous regeneration of hair cells or SGNs in the cochlea.

These approaches, while potentially transformative, require considerable future development, especially given the limitations of the preclinical animal models that are currently available. Specifically, human and animal models do not share the same set of deafness-related mutations. Some of the mutations in mouse do not have orthologous human mutation, such as the *Tmc1* mutation in Baringo mouse which has not been identified in human. A large number of human genetic deafness defects also do not currently have representable transgenic animal models for use in preclinical testing. Furthermore, human and model animals have different development time window, pathogenesis, and recovery physiology. These disparate timelines make it challenging to predict the clinical efficacy of gene and regenerative therapies that have been demonstrated effective in rodent models before the organ of Corti completely degenerates (Wise et al., 2011; Richardson and Atkinson, 2015). For example, efficacy was commonly demonstrated in mice with *in utero* gene transfer (Mianné et al., 2016), or in early postnatal period, because the inner ear development for mice continues until P15. That stands in stark contrast to the development of human ears, which mature before birth (Kopecky et al., 2012).

While the development of effective biomolecular machineries to enable gene replenishment, silencing, and editing are always of strong interests, considerable future development on the delivery vehicle is also needed to realize the potential of gene therapies in clinical practice for treating SNHL. Optimization of viral and non-viral delivery vehicles for safer and more effective targeting to cells in the inner ear is desirable. Furthermore, the designs of these delivery vehicles also need to give consideration for type of cargo they carry. For example, proteins and RNPs have larger sizes than plasmids that small viral carriers, e.g., AAVs, usually cannot accommodate. Lastly, an animal model that matches the optimal therapeutic window in human subjects would greatly improve the success of translation of the aforementioned gene and regenerative therapies.

## Cell Therapy and Delivery Methods

Cell therapy aims to regenerate the inner ear tissues with transplantation of exogenous, stem-cell derived inner ear cells, offering an additional approach for the treatment of non-genetic SNHLs and is currently being investigated in animal studies. Cell replacement may be more attractive over gene therapy for endogenous cell regeneration if there are very few or no surviving cells in the cochlea, e.g., after long periods of acoustic trauma, or if broad activation of cell signaling pathway wish to be avoided.

A major advance in the prospects for cell replacement therapy comes from the successful reprogramming of embryonic stem cells (ESCs) and iPSCs into otic lineages, producing otic epithelial progenitors and otic neural progenitors which can be further differentiated into hair cell-like cells and SGNs (Chen et al., 2012; Boddy et al., 2020). These stem cells and progenitor cells, if successfully engrafted into the desired anatomical locations in the cochlea, could potentially replace the damaged hair cells and SGNs to restore auditory function. Progenitor cells may have the advantage over undifferentiated stem cells for inner ear cell therapy because they possess less tumorigenic capacity (Chen et al., 2018).

Mesenchymal stromal cells (MSCs) have been used in a few clinical cases for managing AIED and were hypothesized to have exerted their effects through paracrine signaling, e.g., with the secretion of anti-inflammatory cytokines, instead of directly differentiating into cochlear cells (Yoo et al., 2015).

Stem cells are often administered directly into the inner ear, with the exception of MSCs which were systemically infused for treating AIED patient. Transplantation of hair cell progenitors is suggested to be best performed with endolymphic injection while neurons are recommended for delivery into the perilymph in the scala tympani. However, given the potential surgical trauma to cochlear tissue with the abovementioned approach, canalostomy was also widely used in animal studies for stem cell delivery (Kanzaki et al., 2020).

### Hair Cell Transplantation

Protocols for hair cell differentiation was first developed *in vitro* with ESCs (Li et al., 2003b) and utricular stem cells (Li et al., 2003a); these generated hair cell-like cells showed several hair cell markers but did not adopt the typical stereocilia morphology. Subsequent strategies using co-culture with chicken stromal cells or conditioned media induced hair cell-like cells which displayed immature, generic stereocilia-like protrusions and produced mechanotransduction response with small currents reminiscent of immature hair cells (Oshima et al., 2010; Ouji et al., 2012). Another strategy using 3D culture differentiated hair cells from mouse ESCs *in vitro* into hair cells carried structural and functional properties comparable to native hair cells, however, they were more similar to vestibular hair cells than cochlear hair cells (Koehler et al., 2013). Another protocol tried to optimize the defined conditions for differentiating hESCs into hair cell-like cells, but they also failed to identify the contributing factors leading to mature *bona fide* hair cells (Ronaghi et al., 2014).

**FIGURE 5:**
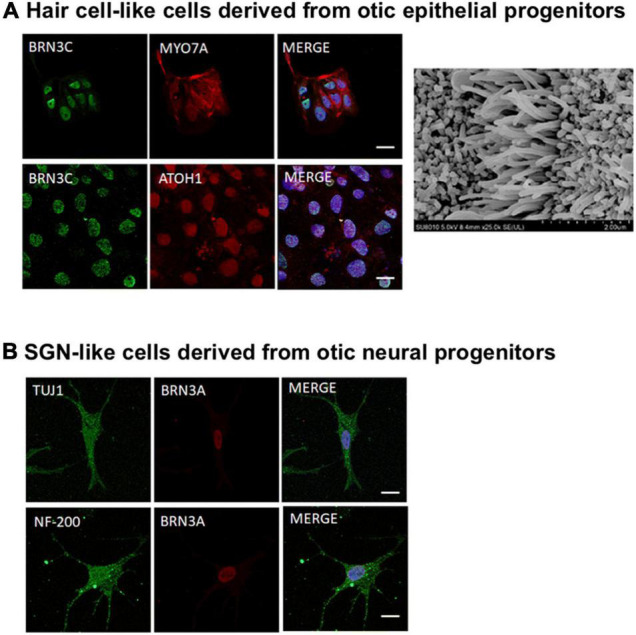
Differentiation of otic progenitor cells *in vitro*. **(A)** (Left) hair cell-like cells derived from otic epithelial progenitors (OEPs) expressed hair cell markers BRN3C (green), MYO7A (red), and ATOH1 (red) (scale bars = 20 μm). (Right) SEM showing apical projections outside hair cell-like cells which are reminiscent of stereocilia (scale bar = 1 μm). **(B)** SGN-like cells derived from otic neural progenitors (ONPs) expressed markers NF200 (green), TUJ1 (green), and BRN3A (red) and formed dendrite-like protrusions (scale bars = 20 μm). **(A,B)** Adapted from Chen et al. (2018), available under Creative Commons license (CC BY 4.0).

Although the differentiation of mature cochlear hair cells has not been reported yet, stem cells and progenitor cells have been used as the candidates for exogenous hair cell replacement *in vivo*. Mouse ESCs have been transplanted into the scala tympani of mice (Chen et al., 2017) or the scala media of guinea pigs (Hildebrand et al., 2005). In both cases, integration of the transplanted cells into the organ of Corti was minimal and change in ABR threshold was non-significant. In another study, human iPSC-induced otic epithelial progenitors (Figure 5A) were transplanted into mouse cochlea through RWM injection (4–5 × 10^5^ cells) (Chen et al., 2018). Some of these cells migrated into the scala media and differentiated into hair cell-like cells in the organ of Corti and formed synaptic connection with native SGNs. However, no hearing response improvement in the treated animals was observed, possibly because of the low rate of cell engraftment, or limited function of these cells. In this study, the protocol used for inducing iPSCs into otic epithelial progenitors also yielded a distinct population of otic neural progenitors which, when further cultivated, were induced into SGN-like cells showing dendrite-like protrusions (Figure 5B). These neural progenitors were not transplanted *in vivo* in this study.

### Neuron Transplantation

In general, stem cell therapy for replenishment of neurons in the inner ear received more interests compared to hair cell replacement therapy early on in scientific endeavors (Hu et al., 2005b; Nishimura et al., 2009), but success in auditory function recovery was still variable.

Mouse ESCs were transplanted, either by itself or with neuronal cografts consisting of dorsal root ganglions, into the scala tympani (through the bony capsule) of guinea pig cochlea. Neuronal cografts significantly enhanced the survival of ESCs compared to mouse ESC-only (*p* < 0.01) in both normal and deaf animals. However, improvement in hearing was not reported (Hu et al., 2005a).

Later on, human ESCs were successfully programmed into otic epithelial progenitors and otic neural progenitors (Chen et al., 2012). When transplanted *in vivo* into a neurophathic deaf gerbil model (where hair cells are preserved), otic neuroprogenitors engrafted into the modiolus of cochlea, formed projections which reached the hair cells in the organ of Corti, and improved ABR response after 4 weeks; mean ABR threshold at 6–38 kHz was 50 dB in treated group compared to 75 dB in untreated group.

In an attempt to improve neuronal differentiation, hESC-derived otic neuronal progenitors were cultured in 3D spheroids for recapitulating the stem cell niche and transplanted, along with BDNF, through RWM injection in diphtheria toxin-deafened mice. The animals were assessed on 90 days after transplantation. Survival of transplanted progenitor cells was marginally low at ∼0.1% and ABR recovery was not observed in the tested animals (Chang et al., 2020).

### Mesenchymal Stromal Cells Therapy

Transplantation of MSCs has also been used to treat autoimmune hearing loss based on their abilities to modulate inflammation and home to sites of cellular apoptosis in the inner ear (Chorath et al., 2020). Success in clinical application has also been reported in using systemically infused MSCs to restore moderate to normal hearing in an adult patient with history of chronic severe hearing loss (Ra et al., 2011). It is believed that MSCs generally do not give rise to new neurons or hair cells, but exert their anti-inflammatory properties and neurotrophic support through paracrine signaling to protect a variety of damaged cells in the cochlea, e.g., neurons, hair cells, fibrocytes, and promote regeneration (Yoo et al., 2015; Kanzaki et al., 2020). MSC labeled with superparamagnetic nanoparticles were developed to improve homing to the cochlea after systemic injection, using a magnetized cochlear implant and external magnet (Le et al., 2017). Magnetically targeted MSCs entered the cochlea and preserved SGN population (80% survival at 4 weeks) and lowered the click ABR thresholds to 75 dB compared to 84 dB in control rats (*p* < 0.05).

### Discussion

Overall, stem cell-based therapy brings the possibility of transplanting exogenous cells into the cochlea to replenish the hair cells and SGNs which are known to have little regenerative capability. Stem cell transplants have shown highly promising results in small animal studies, but they are still at a primitive stage for clinical translation. To date, the mechanism behind the homing and engraftment of stem cells to the organ of Corti remains elusive. Part of the engraftment challenge might be due to the intercellular tight junction in the sensory epithelium which increases the difficulty for cells to entrench, and the high potassium concentration (∼150 mEq/L) in the endolymph which is highly unfavorable for cell survival (Park, 2015). Homing to the target site may be improved by pre-conditioning the microenvironment in the cochlea or the stem cells prior to transplantation (Kamiya, 2015; Peyvandi et al., 2018), thereafter, the new hair cells or progenitors need to restore synaptic connections to the SGNs before they can convey auditory message to the brain. One additional challenge faced by these implanted cells is whether the microenvironment in the cochlea can provide the necessary transcriptional factors, growth factors, or extracellular matrices to support stem cell engraftment and differentiation (Hu and Ulfendahl, 2006).

To this end, cell therapy for SGN regeneration in the cochlea faces similar challenges as those for hair cell transplantation. The survival rate of neural stem cells in the cochlea is far from optimal. In one instance, only 0.4–0.7% of mouse neural stem cells transplanted into deafened guinea pig’s scala tympani survived at 2 weeks post-implantation, and none were found at 4 weeks (Hu et al., 2005b). Once these cells have engrafted, they also need to re-establish neural connections with the auditory system in the brain (Hu and Ulfendahl, 2006). SGN-like cells derived from ESCs were capable of synapsing with cochlear nucleus neurons and forming connections with the central auditory neurons *in vitro* (Liu et al., 2018). As next steps, it would be ideal to observe transplanted neural stem cells or progenitor cells establishing synaptic contacts *in vivo* with hair cells, nearby resident SGNs in the cochlea, and cochlear nucleus cells located in the brainstem.

## Outlook

In this review, we presented recent progress on the therapies and methods of delivery used to treat SNHL. Our discussion was organized based on the three categories of SNHL treatments: pharmacology, gene therapy, and cell therapy which have all been undergoing rapid development in the past two decades. To this end, pharmacotherapy is the most clinically advanced of all three categories while gene and cell-based therapies carry exciting potentials for expanding the spectrum of curable SNHL. As these treatments mature and enter clinical translation, strategies for delivering their cargoes also becomes vital. The next chapter of inner ear therapeutic delivery could benefit from novel strategies to deliver a broad palette of therapeutics, ranging from small molecules to cells, with minimal invasiveness and high targeting precision, thereby simultaneously boosting efficacy and improving safety.

## Author Contributions

SL prepared the manuscript. RY reviewed and edited the manuscript. Both authors contributed to the article and approved the submitted version.

## Conflict of Interest

The authors declare that the research was conducted in the absence of any commercial or financial relationships that could be construed as a potential conflict of interest.

## Publisher’s Note

All claims expressed in this article are solely those of the authors and do not necessarily represent those of their affiliated organizations, or those of the publisher, the editors and the reviewers. Any product that may be evaluated in this article, or claim that may be made by its manufacturer, is not guaranteed or endorsed by the publisher.
